# Molecular cytokine-dependent mechanisms of periodontitis pathogenesis and the potential for pharmacological IL-1 modulation in disease treatment

**DOI:** 10.3389/fdmed.2026.1849807

**Published:** 2026-08-19

**Authors:** Igor Belenichev, Olena Popazova, Oksana Dmytriieva, Sergiy Chertov, Nina Bukhtiyarova, Victor Ryzhenko, Sergiy Oliynyk, Suyeon Lee, Kyu-Ho Yi

**Affiliations:** 1 Department of Pharmacology and Medical Formulation with Course of Normal Physiology, Zaporizhzhia State Medical and Pharmaceutical University, Zaporizhzhia, Ukraine; 2 Department of Histology, Cytology and Embryology, Zaporizhzhia State Medical and Pharmaceutical University, Zaporizhzhia, Ukraine; 3 Department of Propaedeutic and Surgical Dentistry, Zaporizhzhia State Medical and Pharmaceutical University, Zaporizhzhia, Ukraine; 4 Department of Clinical Laboratory Diagnostics, Zaporizhzhia State Medical and Pharmaceutical University, Zaporizhzhia, Ukraine; 5 Department of Medical and Pharmaceutical Informatics and Advanced Technologies, Zaporizhzhia State Medical and Pharmaceutical University, Zaporizhzhia, Ukraine; 6 School of Medicine, Ewha Womans University, Seoul, Republic of Korea; 7 Department of Oral Biology, Yonsei University, Seoul, Republic of Korea

**Keywords:** chronic periodontitis, cytokine receptor modulators, cytokines, IL-1 receptor antagonist, inflammation, oxidative stress, treatment

## Abstract

Periodontitis (P) is one of the most common human dental diseases, with significant medical and social implications. According to the World Health Organization, more than 45% of the adult population worldwide exhibits clinical signs of periodontitis, with 10%–15% of cases becoming generalized with destruction of the alveolar bone. This review presents the results of modern research demonstrating that the etiopathogenesis of periodontitis is not limited to local microbial factors; systemic, social, and behavioral factors can play a significant role, determining the body's susceptibility to chronic inflammation. In this study, we focus on the role of cytokines in the athogenesis of periodontitis. Based on modern research, we demonstratethe general biological significance of cytokines and their role in initiating periodontal inflammation and systemic diseases associated with periodontitis. The molecular and biochemical cytokine-dependent mechanisms of initiation and progression of oxidative stress in periodontitis are covered in detail. The role of individual cytokines in the pathogenesis of periodontitis is shown: interleukin-1 (IL-1), IL-6, tumor necrosis factor (TNF-α), interferon-gamma, IL-10, IL-23, IL-17, and Th17 cells. The review presents a modern approach to complex drug therapy of periodontitis. The arsenal of antiseptics, antibiotics, and synthetic antimicrobial agents available to dentists is presented. A new approach is demonstrated in the use of probiotics—live microorganisms (probiotics, symbiotics, and bacteriophages) that maintain the balance of the oral microbiome. Beyond standard antimicrobial and anti-inflammatory therapies, host modulators, antioxidants, and bioregulatory agents play a vital role in treating P. They effectively suppress proinflammatory cytokines, mitigate oxidative stress, and improve metabolic activity in affected tissues. We present data on the development of individual regenerative structures using bioactive polymers and 3D printing for the future treatment of chronic periodontitis, as well as the potential use of biomaterials with nanostructured components, collagen matrices with nanoparticles, hydrogels with functional peptides, synthetic polymers with a modest antibacterial effect, and transplantation of mesenchymal stem cells, as well as bioactive scaffolds with stem cells. The most important section of the review is devoted to the clinical and pharmacological properties of cytokine modulators—TNF-α blockers, IL-6 blockers, and IL-1 blockers. Using current research results, including our own, we demonstrate the potential of using an IL-1 receptor antagonist and its new dosage form, such as a dental gel.

## 1 Introduction

### 1.1. Analysis of sociomedical and general clinical predictors of periodontitis

Periodontitis is one of the most common human dental diseases, which has a pronounced medical and social character. According to the World Health Organization (WHO, 2023), more than 45% of the adult population of the world have clinical signs of periodontitis, and in 10%–15% of cases, it acquires a generalized form with destruction of the bone tissue of the alveolar process. In Ukraine, according to the National Health Service (2024), the rate of frequency of periodontitis among people of working age is approximately 52%. Consequently, this defines the disease as one of the main causes of premature tooth loss and socioeconomic losses (1–3). Modern studies confirm that the etiopathogenesis of periodontitis is not limited to a local microbial factor. An important role is played by systemic, social, and behavioral factors that determine the susceptibility of the body to chronic inflammation. Therefore, a comprehensive analysis of medical, social, and general clinical predictors that form the individual risk of developing periodontitis and determine the success of its prevention and therapy is relevant (4, 5).

### 1.2 Literature search methodology

The research methods of this study included bibliosemantic, analytical, logical, and generalization methods. We queried major biological and biomedical databases—including MEDLINE, EMBASE, PubMed, Web of Science, and Cochrane Central—to locate English-language studies corresponding to our target keywords: periodontitis predictors and pathogenesis, host modulation therapy, inflammation, periodontal microorganisms, cytokines, general physiological and pathological role, the role of cytokines in the regulation of various signaling pathways in inflammatory periodontal diseases, oxidative stress, periodontitis and systemic diseases, non-drug and drug treatments for periodontitis, pharmacological targets of pathogenically based drug therapy for periodontitis, cytokine receptor antagonists, and preclinical and clinical efficacy. During the search process, the authors also scanned the reference lists of all articles to identify additional studies that may have been missed during the initial search. The authors selected articles, monographs by title, and abstracts for analysis, excluding letters, duplicate studies, commentaries, case reports, letters to the editor, conference abstracts, and works that could not be retrieved. All authors independently and voluntarily selected articles, assessed the quality of the data, their presentation, and the consistency of their interpretation with the main idea of the study, and compiled the final list of references. Each individual literary source was analyzed by two authors separately and geographically distant from each other, and in case of disagreement between them about its information value, a third author was brought in. The authors independently analyzed and summarized the search results, conceptualized their presentation in tabular and graphical format, and developed the main points of the review. The authors worked independently without communicating with each other when analyzing and evaluating literary sources. Although the initial selection of sources was based on English-language titles and abstracts, during the subsequent analysis, the authors did not perform an organic selection based on the language of the full-text source, as well as countries and study design. A bibliographic search for this study was conducted in bibliographic databases for the period 2000–2026. The authors, year of publication, study type, sample characteristics, stimulation agents, and primary outcomes of each study were recorded using a standardized form and ordered chronologically. All selected papers were categorized into *in vitro* studies, *in vivo* studies, or clinical trials. The initial search yielded 989 records; after excluding papers that failed to meet the inclusion criteria, a total of 395 studies remained for discussion in this review.

## 2 Medical and social predictors of periodontitis

Periodontitis has a multilevel sociomedical determination, in which socioeconomic, behavioral, and systemic factors act as key predictors of occurrence and progression. The most important medicosocial predictors were low socioeconomic status, insufficient dental literacy, smoking, type II diabetes, obesity, stress, poor working conditions, and urbanization risks. Modification of behavioral and medicosocial factors increases the effectiveness of treatment and prevents relapses. Further research should be aimed at creating digital prediction models that combine medical, behavioral, and social variables (6, 7).

### 2.1 Socioeconomic determinants of periodontitis

Socioeconomic status is one of the strongest determinants of periodontitis. According to research, low socioeconomic status is directly correlated to increased prevalence of periodontitis. Individuals with lower incomes are less likely to visit the dentist, have poorer oral hygiene, and are less likely to receive preventive care (8–10). It has been shown that in patients with low socioeconomic status, progression of periodontitis occurs 1.9 times faster (11–13).

### 2.2 Behavioral predictors and dental literacy

Behavioral factors are among the modifiable predictors of periodontitis. Tobacco smoking is the most significant behavioral risk factor. Smokers have a 2–3 times greater risk of developing generalized periodontitis than non-smokers (14, 15). Alcohol in excessive doses disrupts microcirculation and weakens the immune response (16). Insufficient levels of dental literacy cause the accumulation of biofilm, a major trigger of inflammation (17). It is known that regular preventive visits to the dentist twice a year reduce the risk of generalized periodontitis by almost 40%. Thus, after correction of behavioral habits and their control, remission of periodontitis lasts for more than 24 months. Regular oral care remains a crucial component of periodontitis prevention (18–20).

### 2.3 Age-related features and aging

Periodontitis is an age-related disease. With age, there is an increase in attachment and bone loss. Studies suggest that each decade of age increases the risk of chronic generalized periodontitis by 12%–15% (2). In people over 45 years of age, the frequency of generalized forms increases 2–3 times compared with younger groups. This is due to progressive changes in collagen structures, a decrease in fibroblast activity, and sclerotic changes in periodontal vasculature (21–23).

### 2.4 Gender and hormonal status

Women during periods of hormonal changes (pregnancy, menopause) demonstrate an increased susceptibility to inflammatory gingival lesions because of endocrine-mediated changes in microcirculation and vascular hyperreactivity (24, 25). Men more often show more severe forms of periodontitis, which is associated with hormonal and behavioral differences. In men, the more severe course is associated with a higher frequency of smoking, hypercholesterolemia, and poorer oral hygiene awareness (26, 27).

### 2.5 Medical and systemic predictors

Type 2 diabetes mellitus is the strongest medical predictor, which, through chronic hyperglycemia, advanced glycation end products, and oxidative stress, increases bone resorption (28–30). According to the American Diabetes Association (399), 60%–70% of patients with type 2 diabetes mellitus have increased bone resorption due to chronic hyperglycemia, advanced glycation and products, and oxidative stress (31, 32). Hyperglycemia promotes glycation of connective tissue proteins, increases the production of metalloproteinases (MMP-8), and reduces the regenerative potential of the gums. Hyperglycemia leads to diabetic glycosylation of proteins, impaired microcirculation, oxidative stress, and inhibition of phagocytosis (33). In turn, periodontitis increases insulin resistance through the release of cytokines IL-6 and tumor necrosis factor-α (TNF-α), which creates a “vicious circle” of mutual influence (34). Another important systemic factor is cardiovascular disease. The “vascular inflammation” hypothesis suggests that *Porphyromonas gingivalis* bacteria can penetrate the vascular endothelium, activating systemic inflammation and the formation of atherosclerotic plaques (35, 36). Obesity and metabolic syndrome are associated with increased levels of IL-6 and TNF-α, which accelerate periodontal destruction (37, 38). It was noted that patients with Body Mass Index >30 had 1.8 times greater average pocket depth (PD) than in the control group. Among the diseases of the gastrointestinal tract, chronic gastritis, gastroesophageal reflux disease, and dysbacteriosis are particularly significant. They change the acid-base balance of saliva, disrupting the settlement of normal oral microflora, which creates conditions for colonization by pathogens (39, 40). Patients with hypertension have a higher incidence of deep periodontal pockets even in the absence of other risk factors (41).

Immunometabolic disorders are decisive in the formation of susceptibility to periodontitis. An imbalance between pro- and anti-inflammatory cytokines, excessive activity of neutrophils and macrophages, deficiency of secretory IgA, or suppression of cellular immunity enhance tissue destruction. In addition, oxidative stress and impaired antioxidant defense play a role, which activate the apoptosis of periodontal cells. Individual susceptibility to periodontitis is largely determined by genetic factors. Polymorphisms of the IL-1β, TNF-α, interleukin-10 (IL-10), and TLR4 genes are associated with increased susceptibility to infectious inflammation (42–44). In addition to genetic modifications, epigenetic modifications play an important role in DNA methylation, histone acetylation, and microRNA regulation. They mediate the influence of environmental factors, such as smoking, stress or nutrition, on the expression of immune response genes (45, 46). Deficiency of vitamins C, D, calcium, zinc, and omega-3 fatty acids negatively affects the condition of the gums and regeneration processes. In patients with vitamin D deficiency (less than 20 ng/mL), a decrease in alveolar bone density by 12% was observed compared with the control group (47, 48).

### 2.6 Environmental and urbanization factors

High levels of air pollution—PM2.5, nitric oxide metabolites (NO*_x_*), and heavy metals—are positively correlated to the intensity of periodontal inflammation. For example, residents of large industrial regions have a 15%–18% higher prevalence of periodontal disease than control populations. In addition, urbanization is accompanied by stress, irregular eating, and low physical activity, which create a metabolic environment for the progression of inflammation (49–51).

### 2.7 Professional factors

Workers in the metallurgical, chemical, and mining industries have a 1.5–2 times higher frequency of periodontitis (52–54). Office workers have a different risk pattern—physical inactivity, stress, and overwork—which also reduces the resistance of periodontal tissues (55, 56). Elevated cortisol and adrenaline levels lead to immunosuppression, decreased neutrophil activity, and increased periodontal inflammatory response. It was found that patients with high levels of anxiety on the Hospital Anxiety and Depression scale had periodontal PDs that were 0.7 mm greater than those in the control group without chronic stress (57, 58).

### 2.8 General clinical predictors of periodontitis

Clinical predictors are objective parameters that reflect the current state of the periodontium and allow to predict its dynamics (59). These include PD, clinical attachment loss (CAL), bleeding on probing (BOP), tooth mobility, and degree of bone loss. Combined with systemic data (age, sex, diabetes, smoking, obesity), they form the basis for risk stratification (60–62).

#### 2.8.1 Periodontal pocket depth

One of the main clinical parameters for assessing periodontal status is PD—an indicator that reflects the loss of epithelial attachment and the level of connective tissue destruction. In recent years, PD has acquired the status of a predictor of the risk of developing and progressing periodontitis. An increase in pocket depth indicates the activity of destructive processes in the periodontium (63–65). The mechanical depth of the pockets directly correlates with the number of microorganisms in the biofilm, the concentration of cytokines (IL-1β and TNF-α), and the level of metalloproteinases (MMP-8, MMP-12, MMP-9), which contribute to collagen degradation. Thus, PD reflects not only the morphological status but also the biochemical status of the disease (66, 67). It has been shown that patients with PD ≥6 mm have a 2.5-fold higher risk of periodontitis progression than those with PD ≤4 mm (68). A machine learning model has been created to predict response to non-invasive periodontitis therapy (69). The most important factor determining the success of treatment is the initial pocket depth (impact factor 0.42; *p* < 0.01). In clinical practice, a PD value ≥5 mm is considered a cutoff value, indicating the need for in-depth sanitation or surgical intervention. The depth of periodontal pockets is not an isolated parameter—its dynamics depend on systemic and behavioral factors. PD assessment is a key criterion for the effectiveness of treatment. A reduction in pocket depth after Scaling and Root Planing (SRP) by 1–2 mm indicates successful stabilization of inflammation (70, 71). It was found that patients who had pockets >5 mm after treatment had a 3-fold higher risk of recurrence within the next three years. In the practice of supportive periodontal therapy (SPT), PD is used as the main indicator to monitor the stability of results. The latest digital technologies, including 3D imaging and artificial intelligence, allow automated measurement of pocket depth and formation of an individual risk profile (72–75).

#### 2.8.2 Clinical attachment loss

CAL reflects the cumulative loss of supporting structures—epithelial attachment, connective tissue fibrous apparatus, and alveolar bone. That is why this indicator is considered the gold standard in the diagnosis and prognosis of periodontitis (76, 77). Attachment disruption is a consequence of the destruction of periodontal ligament fibers under the influence of inflammatory cytokines (IL-1β, TNF-α, and IL-6), metalloproteinase activity (MMP-8 and MMP-9), and osteoclastic bone resorption (78–80). The dynamics of CAL—that is, the gain or loss of attachment—is a direct marker of progression or remission of Chronic Generalized Periodontitis (CGP) (81). CAL measurement is a mandatory element of all international classifications of periodontitis. In modern European Federation of Periodontology (EFP) and AAP protocols, the degree of attachment loss determines the stage of the disease (I–IV) and the level of risk of progression (82). In clinical practice, CAL changes allow to diagnose the stage of the disease (loss of 1–2 mm corresponds to a Stage I, more than 5 mm to severe) to assess the effectiveness of treatment (an increase in CAL by ≥1 mm after therapy indicates positive regeneration) and to predict the outcome (a persistent decrease in CAL is an indicator of stable remission). With the introduction of digital periodontography and 3D scanning, accurate monitoring of attachment dynamics has become possible (82–84).

#### 2.8.3 Bleeding on probing

Bleeding is a marker of active inflammation. Today, BOP is considered not only as a diagnostic sign but also as a prognostic marker of disease activity (70, 85, 86). Bleeding is a consequence of inflammatory damage to the vascular endothelium, increased vascular permeability, and degradation of the extracellular matrix under the influence of mediators (IL-1β, IL-6, TNF-α, and PGE2) (87). Damage to the pocket epithelium leads to a thinning of the barrier, and microtrauma during probing causes the release of red blood cells. The intensity of bleeding directly correlates with the activity of polymorphonuclear leukocytes and the number of pathogenic bacteria, in particular, *P. gingivalis* and *Tannerella forsythia* (40). BOP is one of the simplest and most informative clinical tests. It has high sensitivity (80%–90%) for active inflammation but moderate specificity, as its absence almost excludes active disease and its presence does not always indicate loss of attachment (88). It has been shown that the absence of bleeding for 12 months is associated with a low risk of progression of periodontitis (<10%). In contrast, the presence of persistent BOP in ≥30% of the sites increases the risk of attachment loss by 4–5 times. It has been emphasized that the combination of BOP >20% and pocket depth ≥5 mm has the highest prognostic accuracy for identifying active sites (89–91). Patients with a BOP >25% after therapy have a 3-fold higher risk of relapse within two years (92–94).

#### 2.8.4 Radiographic alveolar bone loss

Radiographic signs of bone loss are a structural marker of chronicity. Patients with defects greater than 30% of the root length have a significantly worse prognosis. The latest digital algorithms [artificial intelligence (AI)-analysis of radiographs] allow for quantitative assessment of bone loss and prediction of further changes (95–97).

#### 2.8.5 Tooth mobility

Increased mobility is a late clinical marker of destructive processes, but its dynamics may reflect the response to therapy. Patients with preserved tooth stability after 6 months of treatment have a better long-term prognosis (98, 99).

## 3 The role of microbiological factors in the pathogenesis and prognosis of periodontitis

**Figure 1:**
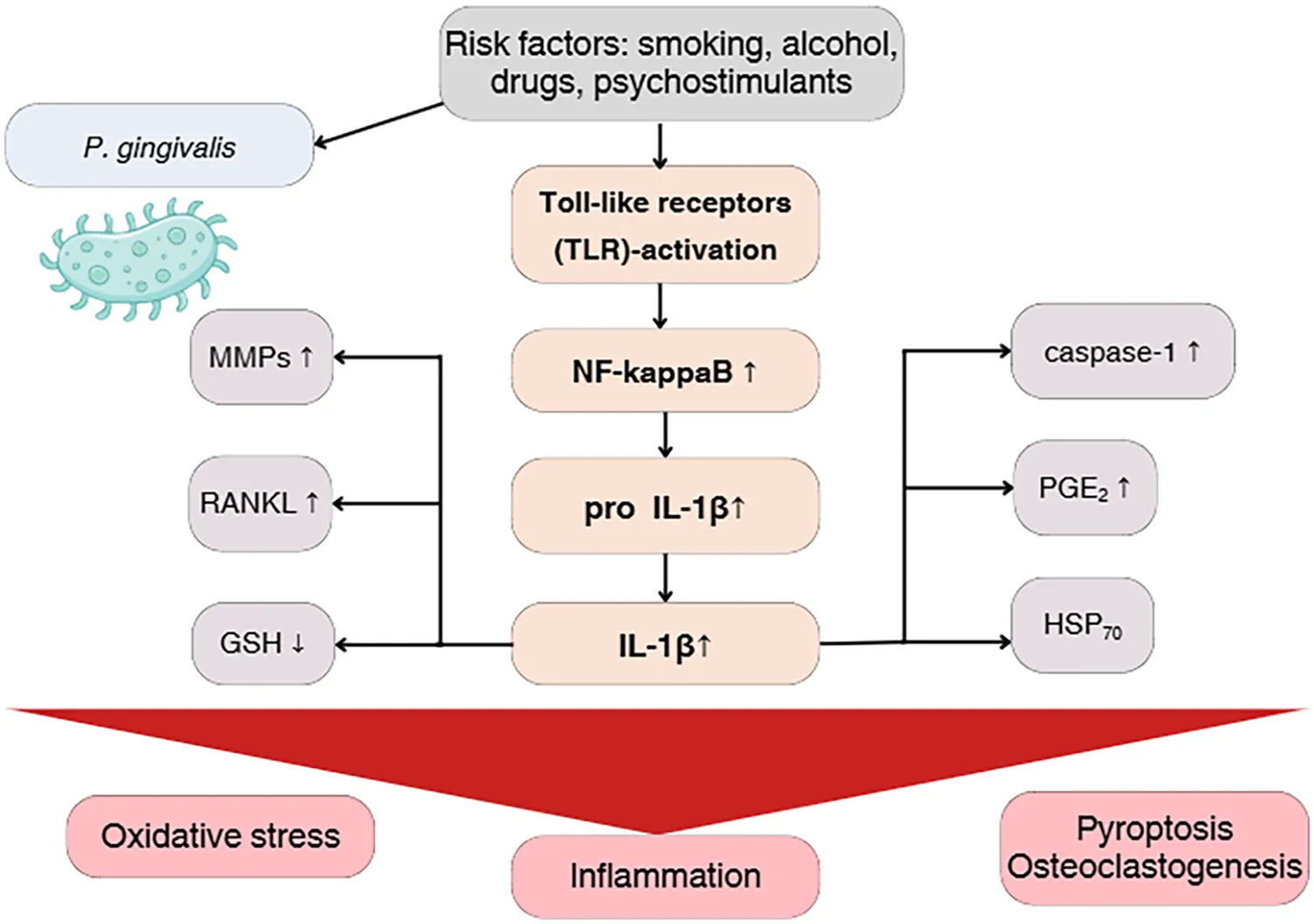
A schematic of IL-1β-dependent signaling in the pathogenesis of periodontitis. Risk factors (smoking, alcohol, drugs, psychostimulants) promote *P. gingivalis* colonization, which activates toll-like receptors (TLRs), triggering caspase-1, NF-κB, and pro-IL-1β upregulation. Subsequent IL-1β maturation drives MMP and RANKL expression, depletion of HSP70 and GSH, and induction of pyroptosis and oxidative stress—collectively promoting inflammation and osteoclastogenesis.

Microbiological studies in recent years have allowed us to identify a number of key species associated with the progression of periodontitis, including *P. gingivalis*, *T. forsythia*, *T. denticola,* and *Aggregatibacter actinomycetemcomitans*. The microbial etiological complex (the so-called red complex—*P. gingivalis*, *T. forsythia*, and *T. denticola*) is recognized as the main microbial predictor of the development of periodontitis (Figure 1) (100, 101).

These bacteria are characterized by high proteolytic activity, the formation of volatile hydrogen sulfide compounds, and the ability to destroy intercellular connections of the epithelium. *P. gingivalis* produces gingivitis enzymes that degrade collagen and components of the immune system, in particular IgG. *T. forsythia* is associated with the formation of deep periodontal pockets, and *T. denticola* has a cytotoxic effect on epithelial cells. The composition of the microbiota in healthy patients differs significantly from that in patients with chronic periodontitis. In healthy periodontium, gram-positive facultative anaerobes (*Streptococcus sanguinis* and *Actinomyces naeslundii*) predominate, while in pathological conditions, gram-negative anaerobes (*Prevotella intermedia*, *Fusobacterium nucleatum*, and *P. gingivalis*) dominate (102–104).

Periodontal pathogens have the ability to form biofilms, complex microbial communities that are protected from the effects of antibiotics and immune cells (105, 106). Microorganisms cause the activation of Toll-like receptors on gingival cells and stimulate the production of proinflammatory cytokines (IL-1β and TNF-α) and collagen-degrading enzymes. This triggers a chain reaction of inflammation that leads to loss of attachment (107). A quantitative analysis of microbial load allows to predict the risk of progression of chronic periodontitis (108, 109). The state of the oral microbiome is closely linked to systemic conditions. Patients with diabetes, obesity, or immunosuppression have a higher colonization of anaerobes and lower microbial diversity (110, 111).

*P. gingivalis* fimbriae play an important role in inflammation and the pathogenesis of periodontitis. Fimbriae could utilize Toll-like receptors (TLR1 or TLR6) for cooperative TLR2-dependent activation of transfected cell lines, which is necessary for the release of NF-κB dimers and, consequently, promotes pro-IL-1β expression. Subsequently, binding of eATP to the P2X7 ion-gated channel receptor is required for IL-1β maturation. Human gingival fibroblasts have a specific response to *P. gingivalis* during IL-1β maturation. Hypoxia can enhance IL-1β maturation. Clinical and experimental data confirm that periodontitis leads to excessive production of IL-1β both locally (in gingival fluid and saliva) and systemically (in blood serum). Osteoclastogenesis is heavily promoted by IL-1β through the upregulated secretion of MMP, RANKL, PGE2, IL-6, and IL-8. Mechanistically, IL-1β drives the expression of matrix metalloproteinases (MMPs); these collagenolytic enzymes degrade the extracellular matrix, directly precipitating bone resorption and tissue destruction. Within fibroblast cells, IL-1β drives the synthesis of PGE2, which subsequently upregulates RANKL expression. Concurrently, IL-1β elevates CX3CL1 levels to mediate the migration of osteoclast precursors beneath the osteoblastic layer, ultimately culminating in osteoclastogenesis. Upon IL-1β exposure, periodontal fibroblasts release proinflammatory mediators such as IL-6 and IL-8, both documented stimulants of bone resorption. At the molecular level, IL-1β activates key signaling cascades—including the NF-κB and mitogen-activated protein kinase (MAPK) pathways—which induce reactive oxygen species (ROS) generation through enzymes like NADPH oxidase. This oxidative stress contributes to a systemic glutathione deficiency that directly aggravates the severity of the inflammatory process. Furthermore, IL-1β triggers the upregulation of iNOS and iNOS mRNA expression alongside this heightened ROS production. Furthermore, oxidative stress drives the chronicity and intensity of inflammatory responses, thereby accelerating pathological tissue destruction and exacerbating the progression of periodontal diseases. Excessive IL-1β levels produced in inflamed periodontal tissues can induce HSP70 expression and reduce endogenous cytoprotection. Thus, IL-1β promotes the secretion of proinflammatory factors and oxidative stress against the background of a decrease in cytoprotective and antioxidant mechanisms, which stimulates osteoclastogenesis and tooth loss.

### 3.1 Smoking

Smoking increases *P. gingivalis* and decreases *Lactobacillus.* Conversely, regular use of probiotics (*Lactobacillus reuteri*) reduces pathogenic bacteria and maintains microbial balance (112, 113).

### 3.2 Modern diagnostics

Modern diagnostics are based on polymerase chain reaction (PCR), metagenomic sequencing, and real-time quantitative PCR (qPCR), which allow the identification of even minimal concentrations of pathogens (114, 115). The use of molecular technologies makes it possible to provide a personalized approach to therapy, taking into account the specific bacterial profile of the patient. An analysis of current sources shows that general clinical predictors are critically important for understanding the pathogenesis and dynamics of periodontitis. They allow not only to assess the extent of the lesion but also to predict the outcome of therapy. The combination of local and systemic indicators provides the greatest diagnostic accuracy. In particular, the combination of PD >6 mm, stable bleeding, and uncontrolled diabetes is a prognostically unfavorable combination (116, 117).

### 3.3 Modern prediction models

A promising direction is the creation of integrated diagnostic and prognostic platforms that automatically analyze the patient's clinical data to form an individual risk map (118). The use of the Random Forest machine learning model enables effective prediction of treatment outcomes. Key factors with the highest prognostic value include initial periodontal pocket depth (PPD), BOP, and IL-1β concentration in gingival fluid or saliva. In one study, the model achieved an accuracy of 0.87 (AUROC) (119). Limitations: Models often predict positive response to treatment more accurately than non-response.

## 4 The role of cytokines in the pathogenesis of periodontitis

Oral health and the etiology of diseases such as chronic generalized periodontitis are profoundly influenced by dental plaque, a continuously proliferating microbial biofilm on the tooth surface (120, 121). More than 30 years ago, it was established that periodontitis is the result of an imbalance in the general microflora due to environmental degradation and declining food quality, which leads to the growth of periodontally pathogenic bacteria (122). Subsequent investigations, however, demonstrated that chronic periodontitis is fundamentally driven by an imbalance among the constituent microbial taxa of the commensal human oral microflora. This dysbiosis serves as a primary driver of localized periodontal inflammation. Consequently, a novel etiological framework was introduced: the “inflammation-mediated polymicrobial emergence and dysbiotic exacerbation” (IMPEDE) model. Within this framework, inflammation is viewed as a direct consequence of dysbiotic shifts occurring throughout the disease course, steering the transition from a state of oral health to active periodontitis (123–125). Historically, various classification schemes segregated periodontitis into two distinct clinical presentations: chronic and aggressive (126). Aggressive periodontitis was characterized by rapid progression, profound tissue destruction, and early-onset edentulism. Conversely, chronic periodontitis was described as a more indolent disease manifestation characterized by pronounced gingival inflammation and substantial polymicrobial colonization across the affected root architecture (5).

In contemporary clinical practice, aggressive and chronic subcategories are merged into a unified diagnosis of periodontitis, which is evaluated using a comprehensive staging and grading framework. This modern classification delineates four distinct stages (stages I–IV) based on the absolute severity and therapeutic complexity of the presentation. Severity metrics are derived from maximal interdental clinical attachment loss, radiographic bone resorption, and secondary tooth loss, while structural complexity is mapped through maximum probing depth and specific osseous defects. The diagnostic profile also integrates the overall extent and anatomical distribution of the disease, classifying it into localized, generalized, or molar–incisor phenotypes. The periodontium itself operates as a dense collagenous matrix anchoring the dentition to the underlying alveolar bone to maintain essential oral function and aesthetics (127, 128), with the disease representing a continuous pathological spectrum starting from healthy, uninflamed tissue and culminating in advanced periodontitis (129). Although early-stage periodontitis primarily impacts the gingival soft tissues, advanced forms of the disease extend beyond the gums to compromise the underlying osseous structures supporting the dentition. This progression into a severe stage is characterized by intense localized inflammation, alveolar bone involvement, periodontal abscesses, and progressive bone resorption (63). To objectively evaluate the severity and complexity of advanced periodontitis, clinical assessments rely on specific diagnostic parameters, including radiographic bone loss, secondary tooth loss, maximum probing pocket depths, interdental attachment levels, and CAL (130, 131).

Dynamic changes in laboratory and clinical parameters of periodontitis can also be prognostic factors for disease progression and the effectiveness of non-drug or drug therapy. Clinical, laboratory, biochemical, and molecular genetic parameters characterizing the severity of periodontitis can indicate potential risks of cardiovascular disease, endocrine disease, digestive system disease, and a predisposition to malignant neoplasms (132–134).

The 2017 World Workshop on the Classification of Periodontal and Peri-Implant Diseases and Conditions resulted in a new classification of periodontitis characterized by a multidimensional staging and grading system. (https://www.perio.org/2017wwdc).

Stage 1 (initial) periodontitis is characterized by a CAL of 1–2 mm and radiographic bone loss limited to the coronal third (<15%). At this stage, there is mild to moderate gingival inflammation and bleeding, with probing depths up to 4 mm and no tooth loss due to periodontitis.

Stage II (moderate) periodontitis is periodontitis is defined by a CAL of 3–4 mm and radiographic bone loss within the coronal third (15%–33%). It is characterized by significant evidence of disease progression, with probing depths up to 5 mm and predominantly horizontal bone loss.

Periodontitis stage 3 (severe) is characterized by deep periodontal pockets (≥6 mm clinical attachment), loss ≥5 mm, and radiographic bone loss extending to the middle or apical third of the root. At this stage, tooth mobility (grades I–II) and the loss of up to four teeth due to periodontitis are observed, often accompanied by vertical bone loss and furcation involvement. Treatment requires a comprehensive approach, including scaling, deep cleaning of the pockets, medication, and possibly surgery and splinting.

Periodontitis stage IV (advanced) is characterized by the same clinical parameters as stage III but with a significant loss of five or more teeth, leading to masticatory dysfunction and severe tooth mobility (grades II–III), and pus. At this stage, severe pain, swollen gums, and general discomfort are observed, and treatment requires complex surgery, often followed by extensive prosthetics, as preserving the teeth becomes problematic. Smoking, poor oral hygiene, and diabetes are important factors contributing to the progression of periodontitis (63, 135–137).

### 4.1 Cytokines—general biological significance

Cytokines comprise a diverse group of low-molecular-weight regulatory proteins, generally spanning a mass range of 6–70 kDa. Synthesized by both lymphoid and non-immune cell types, these molecules serve as essential regulators of tissue homeostasis and immune surveillance (138, 139). Operating within localized microenvironments as well as through the systemic vasculature, cytokines mediate critical physiological pathways, such as intercellular signaling, hematopoiesis, and host defense mechanisms, making them vital indicators of overall health status (140). Furthermore, they orchestrate adaptive physiological responses against pathogens, malignancies, foreign tissue elements, and inflammatory stimuli (141). A defining feature of cytokines is their pleiotropy, characterized by the ability to target diverse cell types simultaneously or sequentially, thereby inducing varied physiological outcomes. Consequently, an individual cytokine can stimulate multiple immune and structural cells to trigger complex downstream biochemical and molecular cascades. Pathological states are frequently characterized by aberrant cytokine expression and shifted concentrations, which often dictate clinical severity and therapeutic resistance (142, 143).

For instance, upregulated TNF-α production drives the pathogenesis of several chronic inflammatory conditions, including psoriasis, periodontitis, and rheumatoid arthritis; correspondingly, targeted inhibition of TNF-α using neutralizing antibodies has proven clinically effective in managing these disorders (144, 145). Increased production of proinflammatory cytokines has been shown to be directly related to organ failure and death. One illustrative example is the so-called cytokine storm, which has been shown to be associated with a poor prognosis in critical cases of coronavirus disease 2019 (146, 147). Ultimately, quantified cytokine profiles serve as valuable biomarkers for immune cell modulation and hold profound clinical utility, particularly as targeted pharmacological interventions aimed at cytokine expression are integrated into multimodal treatments for inflammatory-driven pathologies such as asthma, rheumatoid arthritis, inflammatory bowel disease, periodontitis, and psoriasis (148, 149).

### 4.2 The relationship between periodontitis and systemic diseases

Robust evidence from both experimental models and clinical investigations indicates that periodontitis can elevate systemic concentrations of proinflammatory cytokines, thereby inciting or exacerbating various inflammatory, degenerative, or neoplastic processes throughout the body (150, 151). Experimental studies have shown that modeling periodontitis leads to significant (several times higher than in healthy animals) levels of IL-1β and TNF-α in the blood, indicating systemic inflammation caused by periodontitis. Some of these studies found that high levels of proinflammatory cytokines in experimental chronic periodontitis triggered inflammatory reactions in the brain, subsequently exacerbating pathology similar to Alzheimer's disease. Thus, in rats with chronic periodontitis, modeling Alzheimer's disease led to more severe impairment of cognitive and monistic functions of the central nervous system and contributed to more active neurodegeneration, enhancing neuroapoptosis (152–154). Furthermore, modeling arthritis in experimental animals with periodontitis resulted in more severe morphological consequences, along with higher IL-1β and TNF-α levels in the blood, compared with the group of animals with experimental arthritis alone. Thus, modeling chronic periodontitis with *P. gingivalis* infection affected the clinical and morphological signs of experimental arthritis induced by injection of bovine serum albumin (155, 156).

In groups of animals with experimental arthritis alone, no resorption was observed, while in the group in which, in addition to experimental arthritis, periodontitis was modeled by exposure to *P. gingivalis*, higher levels of TNF-α, IL-1β, and IL-17 were observed, as well as greater joint damage than in control mice. Therapeutic intervention using IL-17RA and IL-1βRA significantly ameliorated symptoms in rats with dual pathologies, confirming that periodontitis initiates cytokine-mediated cascades that worsen arthritis. An alternative mechanism through which periodontitis modulates systemic inflammation involves IL-1β activity (157, 158). Experimental models reveal that periodontitis triggers maladaptive trained myelopoiesis driven by elevated IL-1β concentrations. This aberrant myelopoiesis subsequently predisposes the host to heightened experimental arthritis severity, indicating that the localized inflammatory microenvironment of periodontitis can provoke systemic sequelae and significant comorbidities throughout the organism (151, 159).

Consequently, managing periodontitis represents a potential avenue for mitigating other inflammatory states. Clinical data show that integrating nonsurgical periodontal treatment with standard dermatological therapies provides an additive benefit, significantly lowering psoriasis prevalence and severity (160). In oncology, periodontitis has been shown to increase circulating IL-6 levels and elevate regulatory T-cell counts. This implies that periodontal disease may favor cancer progression by promoting IL-6-dependent neoplastic proliferation while simultaneously suppressing anticancer immunity through regulatory T-cell recruitment (161). Notably, this relationship is bidirectional: systemic, cytokine-mediated inflammation also exacerbates periodontal tissue destruction. Patients with rheumatoid arthritis exhibit a heightened susceptibility to periodontitis, a phenomenon closely tied to elevated TNF-α and IL-1β levels within systemic circulation and the gingival crevicular fluid (162).

### 4.3 Cytokines’ role in initiating the inflammatory process in the periodontium

To preserve tissue homeostasis within healthy oral mucosa, a dynamic equilibrium is maintained between cytokines possessing pro- and anti-inflammatory properties (163). However, the development of gingivitis and periodontitis leads to elevated levels of proinflammatory cytokines inside the affected tissue, shifting this balance toward localized inflammation (164). Interestingly, the expression of these proinflammatory cytokines during periodontitis can be even more pronounced than in other chronic conditions, including inflammatory disorders of the respiratory and digestive systems (165, 166). This phenomenon is clearly illustrated in clinical cases where patients present with both periodontitis and inflammatory bowel disease; in these individuals, the expression of IL-1β and TNF-α is significantly higher in gingival tissue than in the intestinal mucosa. Such findings indicate that high cytokine concentrations within inflamed periodontal structures directly drive local inflammatory responses and tissue fibrosis (167, 168). Beyond managing soft tissue changes, cytokines also regulate bone resorption within the periodontal architecture. Specifically, they control the production of receptor activator of NF-kappaB ligand (RANKL). This specific ligand is secreted by fibroblasts, osteoblasts, and lymphocytes, and it triggers osteoclast activation through direct RANKL/RANK molecular pathways to cause alveolar bone resorption (169).

This pathway is physiologically inhibited by osteoprotegerin (OPG), which competitively binds to RANKL and disrupts the crucial RANKL/RANK interaction (170). Consequently, cytokines govern key pleiotropic pathways vital for maintaining tissue and bone homeostasis within the periodontal microenvironment. An imbalance between pro- and anti-inflammatory cytokines—primarily characterized by the overexpression of IL-1β and TNF-α—occurs through the active involvement of periodontal pathogens alongside patient-specific traits. The contributing roles of gram-negative bacteria, genetic predispositions, behavioral factors (such as smoking, alcohol consumption, psychostimulants, and illicit drug use), and specific pharmaceuticals are well documented (171, 172). These factors collectively initiate molecular-biochemical proinflammatory cascades that drive localized inflammation and subsequent alveolar bone resorption (173).

Microorganisms actively infiltrate and compromise periodontal tissues, generating cytotoxic metabolites such as hydrogen sulfide and ammonia. They further recruit and activate immune populations (including macrophages, dendritic cells, and lymphocytes) alongside resident non-immune cells such as fibroblasts, epithelial cells, and osteoclasts (174, 175). This cellular activation is triggered either by bacterial products engaging Toll-like receptors on target cells or by specific bacterial antigens (176), prompting the robust synthesis of soluble mediators, including cytokines and chemokines.

In periodontitis, cytokines propagate the inflammatory and destructive cascade through several distinct mechanisms: first, they stimulate innate and adaptive immune cells, driving their proliferation and amplifying proinflammatory cytokine production (151); second, they upregulate RANKL expression and receptor binding while concurrently suppressing OPG, precipitating osseous resorption (177); and finally, they induce tissue-resident cells such as fibroblasts to secrete MMPs. These MMPs—encompassing collagenases, matrilysins, and stromelysins—degrade diverse extracellular matrix proteins, leading to structural tissue breakdown (178). In addition, chemokines function as key soluble regulators in chronic periodontitis pathogenesis by controlling the chemotaxis and trafficking of macrophages and lymphocytes, thereby exacerbating the localized inflammatory response. Synthetically, these insights underscore the central role of cytokines in periodontitis pathogenesis, orchestrating the inflammatory events that culminate in local tissue destruction and alveolar bone loss (151).

However, there are a number of controversial issues and unanswered questions regarding the role of cytokines in periodontal inflammation. The hierarchy of cytokines in the pathogenesis of periodontal inflammation is a contentious issue. Some studies dispute the primary role of IL-1β in periodontal inflammation, assigning a more central role to IL-17, produced by Th17 cells, and interleukin-23 (IL-23), which supports this process. Determining which cytokine initiates destruction in each individual case remains difficult. A contentious issue remains whether periodontitis results solely from the hyperproduction of proinflammatory cytokines or whether a primary “deficiency” of anti-inflammatory factors and endogenous cytoprotective factors is unable to restrain the process. Furthermore, the mechanisms regulating protective/destructive processes in so-called protective inflammation remain unclear. Answering these questions can be complicated by significant discrepancies in the results of studies of cytokine levels in gingival fluid, mixed saliva, and blood and the lack of correlation between them.

### 4.4 Cytokines in the initiation of oxidative stress in periodontitis

Oxidative stress serves as a critical determinant in the pathogenesis of periodontitis (129). As previously noted, the initial phases of the disease involve bacterial metabolic by-products that stimulate host cells to release key proinflammatory cytokines, including IL-1β and TNF-α, which act as primary upstream initiators of this oxidative cascade. IL-1β and TNF-α activate signaling pathways (e.g., NF-κB and MAPK), which lead to the formation of ROS through еnzymes such as NADPH oxidаse. IL-1β аnd TNF-α nоt only initiate but also perpetuate virtually all the consequences of the systemic inflammatory response in periodontitis, contributing to an increased risk of systemic diseases of the cardiovascular, metabolic, digestive, and respiratory systems (179–181). Subsequently, increased ROS production suppresses cellular antioxidant defense. The prooxidant effects of IL-1β and TNF-α are promoted by a deficiency of systemic glutathione, which affects the severity of the inflammatory process. IL-1β and TNF-α also play an independent role, along with ROS, in depleting glutathione reserves and increasing oxidative stress (182, 183).

The activation phase of the inflammatory cascade is critically driven by redox signaling networks, specifically through protein phosphatase inactivation and histone acetyltransferase–mediated histone acetylation, with a prominent role played by the CBP/p300 complex. Under conditions of glutathione depletion, the resulting reversible oxidation of thiol groups within serine–threonine protein phosphatase PP2A, as well as tyrosine protein phosphatases SHP1, SHP2, and CD45, causes their functional inactivation. This inhibitory mechanism typically occurs through the structural formation of intramolecular disulfide bonds. Perturbations in the thiol-disulfide equilibrium and downstream redox signaling critically amplify the inflammatory cascade through the mitogen-activated protein kinase pathway. This signaling network triggers a positive feedback loop, where elevated ROS production drives further cytokine release to sustain chronic inflammation. At the cellular level, ROS-induced oxidative damage to lipids, proteins, and DNA causes profound structural and functional impairments within periodontal tissues. Consequently, this persistent oxidative stress exacerbates the intensity of local inflammatory responses, accelerating pathological tissue destruction and driving advanced periodontal disease progression (184–188).

Despite the abundance of *in vitro* and animal studies on the role of cytokines in initiating oxidative stress and the clear evidence of this role, much remains controversial. For example, it is not fully understood which is the primary source of ROS—periodontal microorganisms or cytokines. Furthermore, pathobiochemical factors associated with inflammatory processes in the periodontium (micronutrient imbalance, xanthine oxidase activity, excess reduced pyridine nucleotides, and endogenous antioxidant deficiency) may also be potential mechanisms for initiating ROS. All this limits our understanding of the temporal sequence: which occurs first: increased cytokines, antioxidant deficiency, impaired iron and other micronutrient homeostasis, activation of xanthine oxidase, or activation of NADPH oxidases. Therefore, despite the clear evidence that IL-1β and TNF-α are potent initiators of oxidative stress in the periodontium, escalating local inflammation into bone destruction, controversial issues lie in the realm of drug therapy, specifically, the timing and location of various antioxidants and their interaction with cytokine receptor blockers.

### 4.5 The rоle of individual cytokines in the pathogenesis of periodontitis

#### 4.5.1 Іnterleukіn-1 (IL-1)

As a major proinflammatory cytokine, IL-1 exerts potent immunoregulatory control over the pathogenesis of chronic periodontitis. Both IL-1α and IL-1β are liberated during cellular injury or upon the activation of immune populations, most notably macrophages. Once in the extracellular compartment, these cytokines undergo proteolytic maturation by enzymes such as caspase-1, functioning as alarmins that orchestrate the recruitment and activation of immune cells expressing the IL-1R1 receptor. Through this mechanism, they modulate innate immunity, inflammasome activation, and adaptive T-cell-mediated immune responses. Clinical investigations consistently substantiate the pivotal role of IL-1 in periodontitis pathology; specifically, distinct IL-1 gene polymorphisms (including the coding variants Lys3, Asn3, and Met256) significantly correlate with increased disease susceptibility. Furthermore, quantitative assessments reveal that IL-1 concentrations within the gingival crevicular fluid are markedly elevated in patients presenting with chronic periodontitis relative to periodontally healthy controls (Table 1) (189–192).

**Table 1:** Effects of pharmacological modulation of IL-1.

Modulation	Type effect	Pharmacodynamics	Preclinical studies	Clinical studies
Negative	Anti-inflammatory (167, 169, 209, 381–387)	Reduction of the systemic inflammatory response, decreased levels of acute-phase reactants, and suppression of the production of other inflammatory cytokines, activation of B and T lymphocytes (especially helper cells), and the release of biogenic amines from basophils and mast cells	Рeriodontitis **Preclinical studies** of parenteral and topical administration of an IL-1β receptor antagonist in rats with a pro-oxidant calcium-deficient model of chronic periodontitis (assessing the reduction of IL-1β, TNF-α, and MMPs in the blood and periodontal tissues) and safety evaluation	Рeriodontitis; **Clinical application** of the IL-1β receptor antagonist in patients with moderate chronic periodontitis (assessing the reduction of IL-1β, IL-6, IL-10, TNF-α, MMPs, and C-reactive protein in the blood and gingival crevicular fluid, as well as the normalization of the blood differential leukocyte count)
Negative	Reduction of pathological catabolism (167, 169, 209, 387)	Reduction of cartilage destruction in osteoarthritis by blocking the activation of synoviocytes and chondrocytes	Osteoarthritis and periodontitis **Preclinical studies** of parenteral and topical administration of an IL-1β receptor antagonist in rats (demonstrating a reduction in MMP-1, MMP-3, and MMP-13)	Osteoarthritis and periodontitis **Clinical application** of the IL-1β receptor antagonist in patients with moderate chronic periodontitis and osteoarthritis [assessing the reduction of MMP-1, MMP-3, and MMP-13; IL-1β, TNF-α, and IL-6, as well as type II collagen degradation markers (CTX-II), the cartilage oligomeric matrix protein, and the YKL-40 protein]
Negative	Effect on hematopoiesis (167, 169, 209, 381–387)	IL-1 blockade reduces excessive stimulation of bone marrow hematopoiesis caused by inflammation	Myeloproliferative diseases **Preclinical studies** of parenteral and topical administration of an IL-1β receptor antagonist in rats (demonstrating the normalization of the blood differential leukocyte count and a reduction in the early release of immature leukocytes into the bloodstream, such as band neutrophils, metamyelocytes, and occasionally myelocytes)	Рeriodontitis **Clinical application** of the IL-1β receptor antagonist in patients with moderate chronic periodontitis (assessing the reduction of toxic granulation of neutrophils and the normalization of the blood differential leukocyte count)
Negative	Reduction of hyperalgesia (167, 169, 209)	Decreased prostaglandin and thromboxane synthesis and modulation of sympathetic fibers through decreased expression of receptors for nerve growth factor and bradykinin	Arthritis, fibromyalgia, and diabetic neuropathy **Preclinical studies** of parenteral and topical administration of an IL-1β receptor antagonist in rats [with chronic periodontitis and streptozotocin-induced diabetes mellitus and assessing the reduction of thromboxane B2 (TXB₂) and 8-isoprostanes in the blood]	Рeriodontitis ** Clinical application** of the IL-1β receptor antagonist in patients with moderate chronic periodontitis
Negative	Endotheliotropic (201, 202, 209, 381–387, 396, 397)	Decreased procoagulant activity, expression of adhesion molecules on the endothelial surface, and normalization of eNOS/iNOS expression	Coronary heart disease **Preclinical studies** of parenteral administration of an IL-1β receptor antagonist in rats [using a doxorubicin-induced model of chronic heart failure, chronic periodontitis, and cerebral ischemia, demonstrating the normalization of eNOS/iNOS concentration and expression, vascular endothelial growth factor (VEGF)/EPCR, and an increase in the density of proliferating endothelial cells in muscular arteries and capillaries]	
Negative	Antiapoptotic (185, 203, 209, 396, 397)	Decreased proapoptotic Bax proteins and increased antiapoptotic Bcl-2 protein, decreased amyloid-induced increase in Fas (a receptor that triggers apoptosis); decreased activation of caspase-3 and caspase-8, which are involved in apoptosis	Myocardial infarction (inhibition of cardiomyocyte apoptosis and adverse left ventricular remodeling); Type 1 and 2 diabetes; chronic periodontitis **Preclinical studies** of parenteral administration of an IL-1β receptor antagonist in rats (using a doxorubicin-induced model of chronic heart failure, chronic periodontitis, and cerebral ischemia, demonstrating the normalization of eNOS/iNOS concentration and expression, VEGF/EPCR, and an increase in the density of proliferating endothelial cells in muscular arteries and capillaries)	**Рeriodontitis clinical application** of the IL-1β receptor antagonist in patients with moderate chronic periodontitis
Negative	Antioxidant (174, 182–185, 209, 381–387, 396, 397)	Reduction of iNOS expression, reactive forms of nitrogen monoxide (peroxynitrite, nitrosonium ion, etc.) and inhibition of nitrosative stress, and activation of NRF2, the main regulator of antioxidant genes	Neurodegenerative pathologies; heart failure; chronic periodontitis **Preclinical studies** of parenteral administration of an IL-1β receptor antagonist in rats (using a doxorubicin-induced model of chronic heart failure, streptozotocin-induced diabetes mellitus, chronic periodontitis, and cerebral ischemia, demonstrating a reduction in the density of cells with signs of apoptosis and annexin V-positive cells, along with an increase in intracellular Bcl-2 concentration and a reduction in caspase-8)	Рeriodontitis **Clinical application** of the IL-1β receptor antagonist in patients with moderate chronic periodontitis (assessing the reduction of caspase-8 and the elevation of Bcl-2 in the gingival crevicular fluid)
Negative	Neuroprotective (152, 153, 180, 181, 209, 396, 397)	Intracellular stress triggers an increase in HSP70 levels in parallel with the activation of the redox-sensitive transcription factors AP-1, NF-κB, and NF-1. This cascade drives the upregulation of key antioxidant genes, namely, glutathione peroxidase, glutathione reductase, and glutathione transferase. In addition, *de novo* biosynthetic pathways are stimulated through the increased expression of genes targeting γ-glutamyl transferase and γ-glutamylcysteine synthetase, enzymes critical for stabilizing the intracellular pool of reduced glutathione	Ischemic strokes; prenatal hypoxia **Preclinical studies** of parenteral administration of an IL-1β receptor antagonist in rats (acute and chronic cerebral ischemia, prenatal hypoxia, assessing the reduction in mortality and neurological deficit via the P. McGraw scale, a reduction in hippocampal neuronocytes with signs of apoptosis, and a reduction in neuron-specific enolase and S-100 protein)	
Negative	Reparative regeneration (204, 209, 381–387)	Acceleration of reparative regeneration by suppressing elevated IL-1 expression, reducing chronic inflammation, and enhancing growth factor activity. Excessive IL-1 suppression may impede regeneration	Diabetic wounds, ligament damage, and chronic periodontitis **Preclinical studies** of parenteral administration of an IL-1β receptor antagonist in rats (chronic periodontitis, streptozotocin-induced diabetes mellitus, and assessing the reduction of IL-1β, IL-6, IL-10, TNF-α, and MMPs, along with the elevation of EGF and VEGF)	Рeriodontitis ** Clinical application** of the IL-1β receptor antagonist in patients with moderate chronic periodontitis (assessing the reduction of IL-1β, IL-6, IL-10, TNF-α, and MMPs, along with the elevation of EGF and VEGF)
Positive	Reparative regeneration (205, 206, 209)	Activation of the NF-κB signaling pathway. Stimulation of fibroblast proliferation and growth factors. Stimulation of stem cells. Increased synthesis of collagen, collagenase, and other enzymes. Formation of hypertrophic or keloid scars	Short-term modulation after trauma and burns **Preclinical studies** of parenteral administration of an IL-1β receptor antagonist in rats following skin wounding (assessing the mRNA expression of c-fos and NF-κB factors)	
Positive	Immunomodulatory (189, 205, 209, 381–387)	Enhanced immune response	Immunodeficiencies or certain types of tumors. The use of agonists is considered a cytokine-based immunotherapy method aimed at overcoming immunosuppression in the tumor microenvironment **Preclinical studies** of parenteral administration of an IL-1β receptor antagonist in rats (chronic periodontitis and assessing its effect on IFN-γ and TNF-α expression)	**Clinical application** of the IL-1β receptor antagonist in patients with moderate chronic periodontitis (assessing its effect on IFN-γ and TNF-α expression)

It is worth noting that excessive pharmacological suppression of IL-1β disrupts the core mechanisms of innate immunity, defense against infections, and tissue repair processes, which may lead to (207–209): (1) The risk of severe infections: Because IL-1β stimulates phagocytosis, neutrophil mobilization from the bone marrow, and T-cell activation, its deficiency induces an immunodeficient state. Patients receiving IL-1 inhibitors (e.g., anakinra or canakinumab) are more susceptible to bacterial and viral infections. (2) Masking of early inflammatory signs: Significant suppression of IL-1β can mask the primary signs of an oncoming inflammation, complicating infection diagnosis. This is driven by the disruption of central, IL-1β-dependent regulatory mechanisms within the hypothalamus. (3) Impairment of T-cell response, antibody production, and vaccine efficacy: This includes a weakened response to immunization and an increased risk of complications when using live vaccines. (4) Impaired reparative processes and wound healing. Excessive pharmacological suppression of TNF-α disrupts the body's baseline defense mechanisms (209, 210): (1) Increased vulnerability to tuberculosis, fungal and serious bacterial infections, as well as viral diseases. (2) Impairment of TNF-α-dependent recognition and elimination of atypical cells, which theoretically increases the risk of certain malignancies such as lymphomas. (3) Induction of autoimmune reactions, including psoriasis, lupus-like syndrome, or demyelinating diseases of the nervous system (similar to multiple sclerosis).

Particularly elevated concentrations of this cytokine are found in elderly patients compared with younger patient groups. However, no statistically significant differences in systemic IL-1 levels have been detected when comparing patients aged 40 years or younger who have chronic periodontitis with those with aggressive periodontitis (193, 194). In cases of chronic periodontitis, IL-1β and the proinflammatory cytokine TNF-α are believed to regulate the extension of the inflammatory front into deeper regions of the connective tissue. Within these areas, they induce the loss of connective tissue attachment, stimulate osteoclast activation, and drive subsequent alveolar bone loss (123, 195). The primary role of IL-1 in mediating periodontal tissue destruction is well documented by research involving various experimental models of periodontitis (including periodontitis caused by periodontally pathogenic bacteria, ligature-induced periodontitis, and periodontitis triggered by prooxidants and calcium deficiency). It was found that in these mumps models, a significant local and systemic increase in IL-1β was observed against the background of signs of periodontal inflammation (196, 197). Experimental data demonstrate that the functional inactivation of the endogenous IL-1 inhibitor, interleukin-1 receptor antagonist (IL-1RA), accelerates disease progression, manifesting as accelerated alveolar bone resorption, increased tooth mobility, and deepened periodontal pockets. Mechanistically, this phenotypic shift is driven by an expansion of IL-17-expressing immune populations alongside an upregulation of IL-17 itself, its associated transcripts (IL-1β, IL-6, IL-23, and TGFβ), and Rank. Collectively, these findings indicate that IL-1RA exerts a critical protective effect within the periodontium by suppressing an IL-1-dependent, hyper-IL-17-mediated inflammatory response in the gingival tissue (198–200).

#### 4.5.2 Interleukin-6

As a pivotal regulatory cytokine, IL-6 modulates chronic inflammatory processes by engaging target cells through the membrane-bound IL-6R receptor. Alternatively, cells lacking this specific receptor can respond to IL-6 through trans-signaling, wherein IL-6 binds to soluble IL-6R to subsequently activate gp130 on target cell surfaces (211). These dual pathways enable IL-6 to mobilize diverse target cell populations within the chronic periodontitis microenvironment. Clinical data show that IL-6 concentrations within the gingival crevicular fluid of chronic periodontitis patients are significantly elevated compared with healthy individuals. In apical periodontitis, local IL-6 expression directly correlates with both the volumetric size of periapical lesions and patient age (212, 213). Conversely, no significant variations were detected in systemic IL-6 concentrations when researchers compared young cohorts (aged 40 years or younger) diagnosed with either chronic or aggressive forms of periodontitis (214, 215). At the periapical site, *P. gingivalis* acts as a key stimulus, triggering macrophage activation and IL-6 production through the STAT3 transcription factor pathway (216). Furthermore, comorbid malignancies exert a synergistic effect in periodontitis patients, markedly compounding serum IL-6 upregulation (217).

The presence of calculus in patients with periodontitis yields higher systemic and salivary concentrations of IL-6 relative to patients with periodontitis alone and healthy controls, with baseline IL-6 levels closely mirroring disease progression (218). Immunohistochemical analyses of advanced periodontal lesions demonstrate the presence of CD4+ T cells that co-express IL-6. Notably, pharmacological management of periodontitis prompts a distinct downregulation of IL-6 within the gingival crevicular fluid, suggesting that this cytokine could serve as a reliable laboratory biomarker for monitoring therapeutic efficacy (219–221). For example, in periodontitis patients presenting with secondary malocclusion, conventional drug regimens decreased both serum and crevicular IL-6 levels following 6 months of intervention. Similarly, in obese patients with comorbid periodontitis, standard drug therapy achieved a comparable reduction in crevicular and systemic IL-6 after 3 months (222, 223). Mechanistically, IL-6 is predominantly synthesized by locally activated immune populations within periodontal lesions, such as neutrophils and macrophages, which maintain the capacity to secrete IL-6 *ex vivo* upon bacterial challenge (224). *In vivo* experimental models validate this high expression within lesions, where multiple cell types—including immune cells and osteoblasts—fuel its synthesis. Operating alongside IL-1 and TNF-α, IL-6 amplifies proinflammatory circuits, mobilizes innate and adaptive immune cells, and drives alveolar bone resorption. Consistently, experimental *P. gingivalis* infection accelerates local IL-6 expression, leading to downstream STAT3 activation and the polarization of M1-like macrophages (151, 216).

#### 4.5.3 Tumоr nеcrosis fаctоr (TNF-α)

As a major proinflammatory cytokine synthesized predominantly by immune cells such as macrophages and lymphocytes, TNF-α exists in both soluble and membrane-bound isoforms. By interacting with its two distinct receptors, designated TNFR1 and TNFR2, TNF-α regulates an array of critical inflammatory pathways. These downstream cascades primarily govern immune cell recruitment, activation, and survival, while simultaneously driving angiogenesis and pathological structural tissue damage (225, 226). Elevated serum soluble TNF-α levels were observed in patients with periodontitis compared with healthy controls and were associated with plaque bacteria (*A. actinomycetemcomitans and P. gingivalis*). Modeling chronic periodontitis results in significant increases in TNF-α in the blood and periodontal tissues of animals, along with deep periodontal pockets and tooth mobility. High TNF-α levels (along with IL-1β) in the patients blood with rheumatoid arthritis are risk factors for periodontitis (227, 228). Non-pharmacological treatment of periodontitis did not suppress TNF-α levels in gingival crevicular fluid, whereas pharmacological treatment of periodontitis reduced TNF-α levels in gingival crevicular fluid and serum after 6 months (229). The function of the TNF-α signaling network was evaluated using TNFR1 knockout mouse models of apical periodontitis triggered by root canal inoculation with *A. actinomycetemcomitans* and *P. gingivalis.* The data demonstrated that TNFR1 deficiency mitigated disease progression—as assessed through clinical and molecular-biochemical markers—confirming that the TNF-TNFR1 signaling pathway operates as a major proinflammatory driver in destructive periodontal diseases (151, 230, 231). TNF-α acts by initiating local inflammatory responses and facilitating their expansion into deeper periodontal layers, which directly promotes the loss of connective tissue attachment and alveolar bone (232).

#### 4.5.4 Interferon-gamma (IFN-γ)

As a potent regulatory cytokine synthesized by immune populations such as lymphocytes, IFN-γ activates target tissues by binding a heterodimeric receptor complex consisting of the IFN-γR1 and IFN-γR2 subunits. These receptor proteins are widely expressed across various host cell types, including macrophages and epithelial cells. Within the periodontal microenvironment, IFN-γ is recognized as a key mediator of proinflammatory events, amplifying localized immune cell recruitment and driving bone remodeling cascades (233–235).

Several clinical studies show a distinct increase in circulating IFN-γ levels during periodontitis. Compared with healthy individuals, patients with periodontitis display higher serum concentrations of IFN-γ, TNF-α, and IL-10. Interestingly, this systemic elevation of IFN-γ tracks with an increase in local plaque accumulation, reinforcing the theory that microbial antigens stimulate its production (197, 236, 237). Data concerning the gingival crevicular fluid remain controversial, with some studies reporting elevated crevicular IFN-γ levels and others finding no significant differences relative to healthy controls. However, tissue-level analyses through immunohistochemistry in advanced periodontal lesions demonstrate that infiltrating CD4+ T cells coexpress IFN-γ, providing evidence for a local Th1 T-cell involvement (238, 239).

Host genetic background also influences this cytokine axis; patients carrying the IFN-γ + 874A/T gene polymorphism exhibit a significantly higher immunohistochemical staining for IFN-γ in interdental gingival tissues than control subjects, which corresponds to a heightened risk of chronic periodontitis and supports its role as a pathological driver. At the cellular level, IFN-γ regulates osteoclastogenesis to promote bone resorption and stimulates macrophages to generate neopterin, which prevents macrophage apoptosis by protecting them from local oxidative stress (240, 241). Animal models show that IFN-γR1 expression is upregulated early during the active initiation phase of experimental periodontitis rather than during later chronic progression, which matches the overall induction of IFN-γ. When challenged with the pathogens *A. actinomycetemcomitans* or *P. gingivalis*, IFN-γ-knockout mice fail to develop the classic clinical signs of periodontitis—such as deep probing depths, increased tooth mobility, spontaneous bleeding, and alveolar bone resorption—that occur in wild-type mice with normal physiological levels of the cytokine. These definitive outcomes underscore the essential role of IFN-γ in driving osteoclastogenesis and maintaining local concentrations of downstream proinflammatory mediators, specifically IL-1 and TNF-α (242–244).

#### 4.5.5 Interleukin-10

Belonging to the IL-12 cytokine family, IL-23 is a heterodimeric mediator comprised of a p19 and a p40 subunit, the latter of which is shared with heterodimeric IL-12 (p35/p40). In the context of inflammatory disorders such as periodontitis, IL-23 acts as an upstream stimulus that prompts innate lymphoid cells and Th17 cells to secrete proinflammatory cytokines, thereby initiating multiple downstream inflammatory pathways (245, 246). The vast majority of clinical investigations document significantly elevated concentrations of both IL-23 and IL-17 within the gingival crevicular fluid of patients presenting with periodontitis or gingivitis relative to healthy control cohorts. Local expression levels of this IL-23/IL-17 axis correlate positively with periodontitis severity and progression, tracking closely with clinical parameters such as probing depth, clinical attachment loss, and the gingival index. Furthermore, the distinct positive correlation between local IL-23 and IL-17 concentrations supports the paradigm wherein IL-23 drives IL-17 upregulation and the clonal expansion of Th17 populations throughout the course of periodontal disease (247, 248).

#### 4.5.6 Interleukin-23

IL-23, a member of the IL-12 cytokine famіly, is a heterodimer composed of p19 and p40 subunits, sharing the latter with IL-12 (p35/p40). Within the pathogenesis of inflammatory disorders like periodontitis, IL-23 initiates multiple downstream inflammatory cascades by stimulating Th17 cells and innate lymphoid cells to secrete proinflammatory cytokines (249, 250). Consequently, clinical data frequently reveal elevated concentrations of both IL-23 and IL-17 within the gingival crevicular fluid of patients suffering from gingivitis or periodontitis relative to healthy controls. This IL-23/IL-17 axis directly correlates with the clinical parameters of tissue destruction, such as probing depth, clinical attachment loss, and the gingival index. The synchronous elevation of these cytokines aligns with the established role of IL-23 in promoting Тh17 cell clonal expansion and driving IL-17 expression during periodontal degradation (251, 252). In рatіents dіagnosed with chronic periodontitis or periodontitis secondary to genetic immunodeficiencies like leukocyte adhesion deficiency type 1, IL-23 is predominantly produced by epithelial cells situated near the periodontal tissues, suggesting that epithelium-derived IL-23 acts as a regulator of disease activity. Mechanistically, IL-23 can stimulate osteoblasts to produce RANKL and accelerate osteoclast maturation, which contributes directly to inflammatory bone destruction (253, 254). However, experiments using p40-deficient mice (lacking both IL-12 and IL-23) showed that following infection with *P. gingivalis*, these animals experienced accelerated tissue breakdown despite having a reduced infiltration of inflammatory cells, indicating that these cytokines are vital for modulating and controlling the overall disease process (255, 256).

#### 4.5.7 Interleukin-17 (IL-17) and Th17 cells

The IL-17 family consists of six proinflammatory cytokines, classified as IL-17A through IL-17F. Within this group, IL-17A and IL-17F are considered central to the development of inflammatory periodontal conditions, including chronic periodontitis. Lymphocytes and innate immune cells, such as innate lymphoid cells, produce both cytokines when stimulated by IL-1β or IL-23 (257, 258). By binding to target cells that express IL-17R, IL-17A and IL-17F manage protective immune responses against fungal infections, drive neutrophil recruitment, and strengthen barrier function at the oral mucosal surfaces (259). Five distinct subunits make up the IL-17R family, specifically IL-17RA, IL-17RB, IL-17RC, IL-17RD, and IL-17RE (260, 261). When researchers conducted a flow cytometric analysis on blood samples from patients with periodontitis, they found a significant elevation in both IL-17A-positive cells and IL-17F-positive cells compared with healthy individuals. In addition, statistical data highlighted a significant correlation between IL-17A-positive cells and clinical parameters of disease severity, including attachment loss and pocket probing depth. These results indicate that these specific IL-17-producing immune cells are directly involved in the development and pathogenesis of periodontitis (151, 262).

Research also demonstrates that patients diagnosed with stage I and II periodontitis exhibit increased salivary IL-17 concentrations compared with healthy controls. Conversely, patients with stage III periodontitis show a marked reduction in salivary IL-17 relative to the control group (263). Additional investigations have detected upregulated IL-17 expression within the gingival crevicular fluid of patients with periodontitis. At the core of the inflamed periodontium, investigators recorded a heavy infiltration of IL-17-producing T lymphocytes, whereas CD68+ macrophages locally produced IL-23, a cytokine known to induce Th17 cells (264, 265). The presence of both IL-17 and IL-23 was identified primarily within periodontal lesions, particularly inside tissues immediately adjacent to areas of bone destruction (266).

The involvement of IL-17 in periodontitis pathogenesis has been evaluated further through experimental models. Data show that ligature-induced modeling prompts a substantial rise in IL-17 concentrations within the blood and periodontium of rats, where IL-10 and IL-1RA act as vital soluble mediators that suppress IL-17 overexpression. For instance, in IL-10-deficient rats in which periodontitis was modeled through *P. gingivalis* infection, researchers observed severe bone loss alongside elevated concentrations of IL-17 and oxidative stress products (267, 268).

Similarly, IL-1RA plays a protective role by limiting IL-17 overexpression in experimental periodontitis. Specifically, IL-1RA -/- knockout animals demonstrated an increased presence of IL-17-producing T cells, which caused severe alveolar bone loss. Administering anti-IL-17 antibodies successfully minimized this alveolar bone loss, confirming that IL-17 is a central regulator of bone metabolism (198, 267). Consequently, IL-17 works alongside IL-1β, IL-6, and TNF-α as a key proinflammatory cytokine in periodontitis capable of triggering osteoclast activation and bone resorption. Furthermore, IL-17 functions as a major driver of neutrophil-mediated tissue inflammation and bone degradation during the disease. Finally, IL-17 expression during periodontitis may aggravate other systemic chronic inflammatory pathologies. In animal experiments, rats with an IL-17 knockout did not develop joint damage or show increased systemic levels of TNF-α and IL-1β when periodontitis was modeled concurrently with an arthritis model (269, 270).

## 5 Modern approaches to the complex therapy of periodontitis

Рeriodontitis is a multifactorial disease, in the pathogenesis of which dysbiosis of the microbial biofilm complex of the oral cavity plays a key role. That is why the main principle of modern treatment is etiotropic therapy, which aims to eliminate pathogenic microorganisms, normalize the microbiocenosis, and prevent relapses. The complex therapy of periodontitis is based on a systemic combination of mechanical, drug, host-modulatory, surgical, and regenerative treatment methods. It aims to eliminate the causes of inflammation, control the microbial factor, restore the tissue structure, and stabilize the results obtained (271–273).

A comprehensive therapy of periodontitis should begin with the stage of patient preparation: motivation, personal hygiene training, and correction of systemic factors (diabetes control, recommendation to quit smoking, and optimization of nutrition and microelement status). The modern concept of etiotropic treatment is based on a combination of mechanical sanitation, pharmacological effects, and biotechnological methods that allow to ensure control of bacterial load, modify the reactivity of the organism, and optimize regenerative processes in periodontal tissues (132, 274). The first stage of treatment is the elimination of the microbial biofilm complex and dental deposits. It includes professional oral hygiene, removal of supra- and subgingival deposits using ultrasonic scalers and Gracey curettes, polishing of root surfaces, correction of occlusion, and elimination of traumatic factors (275). According to the recommendations of the EFP, SRP—manual scaling and root planing are basic components of all periodontitis treatment regimens (126). Scaling and root planing reduce bacterial load by 90%–95%. Meta-analyses show that the effectiveness of SRP depends on the depth of the pockets: for pockets up to 4 mm, complete elimination of inflammation is possible, and for deeper pockets, supplementation with medications is necessary. SRP provides an average reduction in pocket depth of 1.2–1.8 mm and an improvement in clinical attachment by 0.6–1.0 mm. It is important to emphasize that the result depends on the qualification of the clinician, the condition of the root surfaces, and the preparation of the patient (131, 276). In recent years, ultrasonic systems (Cavitron, Vector) have become widespread, which provide delicate cleaning of the root surface and cause less trauma to the gums. The use of ultrasound also increases the effectiveness of antiseptic solutions because of the cavitation effect. However, after 48–72 h without chemical control, the biofilm is restored (277).

Therefore, mechanical treatment should be combined with local antimicrobial agents. Local antiseptics are the basis of etiotropic therapy of the initial and middle stages of periodontitis. First-line drugs include chlorhexidine bigluconate (0.12%–0.2%), cetylpyridinium chloride, povidone-iodine, triclosan, and combined agents with essential oils. Chlorhexidine remains the gold standard, but its long-term use (more than 3 weeks) can cause dysbiosis and taste changes (278). Newer agents such as octenidine dihydrochloride and etheriol complexes have shown similar efficacy with less cytotoxicity (400). Antibiotic-containing gels and microencapsulated systems are useful, such as metronidazole gel (0.25%–1%), doxycycline (10% gel, Atridox), and minoxycycline (Arestin). They provide sustained release of the antibiotic without systemic loading and are used to enhance the effectiveness of SRP, especially in areas with difficult access. Current formulations include chlorhexidine, metronidazole, minoxycycline, clindamycin, and cephalosporins, which are administered as controlled-release gels, films, or capsules (279, 280). For example, the use of a gel with metronidazole or minoxycycline in the pocket cavity after SRP allows maintaining high drug concentrations for a long time and avoiding systemic effects. A multicomponent gel based on chlorhexidine, enzymes, and calcium has been tested in Ukraine, which reduces gingival bleeding by 60% after 4 weeks of treatment. It has been shown that the combination of SRP + local gel gives better results in terms of gingivitis index and pocket depth reduction after 6 months compared with SRP alone (281).

Systemic antibiotic therapy is used in generalized forms or when local treatment is ineffective. One of the common schemes is the use of systemic antibiotics, mainly a combination of amoxicillin and metronidazole. It has been shown that the addition of these drugs to SRP leads to an improvement in clinical parameters (reduction in pocket depth and increase in attachment) by an additional 0.3–0.5 mm. Such therapy should be strictly individualized, because excessive use of antibiotics produces resistance, changes the microbiota, and can cause intestinal dysbiosis and negative systemic effects (282, 283). According to the WHO Global AMR Report (2023), more than 20% of *P. gingivalis* and *F. nucleatum* isolates demonstrate resistance to metronidazole and clindamycin. This necessitates rational antibiotic therapy—prescribing drugs only according to indications, with short courses and strict adherence to dosage (284). It is recommended to avoid the routine use of systemic antibiotics without laboratory confirmation of the severity of the process. The concept of “antimicrobial stewardship in dentistry” has been introduced, which involves minimizing antibiotics and emphasizing local agents, photodynamics, and probiotics. Photodynamic therapy is based on the activation of a photosensitizer (methylene blue and toluidine blue) with light of a certain wavelength with the formation of active oxygen species. As a result, active oxygen species are formed that destroy pathogens and pathogenic microorganisms. Photodynamic therapy does not cause resistance and is minimally traumatic (285, 286). This study confirms that photodynamic therapy reduces the number of *P. gingivalis* by 3–4 times without damaging healthy cells, and its effectiveness is comparable to or higher than that of local antibiotic therapy (287, 288). In Ukrainian conditions, photodynamic therapy demonstrated a positive clinical effect in 78% of patients (289). A new direction is the use of probiotics—live microorganisms (probiotics, symbiotics, and bacteriophages) that maintain the balance of the oral microbiome. *L. reuteri*, *L. rhamnosus*, and *Bifidobacterium longum* have shown the ability to reduce the number of pathogens *P. gingivalis* and *T. forsythia* in periodontal pockets, as well as suppress inflammatory mediators (290, 291).

Local clinicians report that the use of probiotics in combination with standard therapy reduces the bleeding index by 40% and improves clinical adherence—their effectiveness has been proven as an adjunct to mechanical sanitation. Studies are also being conducted in Ukraine on the use of combined probiotic pastes based on *Lactobacillus casei* and plant extracts, which have shown a 25% reduction in the frequency of relapses (292, 293). The current trend is the introduction of adjuvant therapy aimed at modifying the body's response—the so-called host modulation. The pathogenesis of periodontitis is characterized by an excessive immune response of the body, and therefore, it is advisable to use host modulators—drugs that reduce the activity of inflammation without suppressing immunity. The most extensively studied and FDA-approved drug in this class is sub-antimicrobial dose doxycycline (20 mg twice daily) (SDD).

Since the 1960s, with the identification of the role of periodontal bacteria in the pathogenesis of periodontitis, it has been recognized as an infectious disease (now classified as dysbiosis), and antibacterial drugs, including antibiotics, have been widely used in its treatment. In the 1980s, with new advancements in fundamental and clinical medicine, host response mechanisms were identified as mediators of destruction in collagen-rich periodontal tissues, and periodontal pathogens came to be viewed as a “trigger” for the inflammatory/collagenolytic reaction characteristic of actively destructive periodontitis.

Thanks to the work and efforts of Dr. Lorne M. Golub, a new pharmacological strategy emerged, termed “host-modulating therapy.” It was based on two postulates: the ability of tetracycline antibiotics to significantly inhibit periodontal destruction through a previously unknown capacity to inhibit host MMPs (along with other mechanisms, including antioxidant ones), and the ability of non-steroidal anti-inflammatory drugs to reduce the severity of periodontitis. Subsequently, studying the molecular mechanisms of the non-antibacterial action of tetraphenolic chemically modified tetracycline molecules initiated the development of a new category of matrix metalloproteinase inhibitors—biphenolic chemically modified curcumins, which demonstrated high efficacy and safety in preclinical studies.

It was precisely through the work of Dr. Lorne M. Golub that the dose–response relationship of doxycycline was identified. Thus, while standard high doses of doxycycline kill subgingival bacteria, at an SDD of 20 mg per day, the antibacterial effect disappears, and the ability to significantly inhibit MMPs emerges.

An innovative host modulation therapy (HMT) was developed, which was aimed not at eradicating pathogenic periodontal flora but at blocking the excessive immune response, active proinflammatory factors, enzymes, and oxidative stress that destroy the host's own collagen and alveolar bone. By combining various anti-inflammatory agents (with differing mechanisms of action) and antioxidants (both plant-derived and synthetic) with conventional treatments for chronic periodontitis (such as scaling and root planing), HMT aims to limit bone and attachment loss, while promoting tissue healing. SDD serves as the active component of HMT (31, 32, 294). SDD is a widely utilized treatment method for chronic periodontitis, based on its ability to inhibit enzymes that destroy bone and periodontal ligaments. At such a low dosage, it functions not as an antibiotic but as an agent that reduces inflammation. It reduces the activity of metalloproteinases (MMP-8 and MMP-9), which destroy collagen, without affecting the bacterial flora. Other studies have also confirmed that the inclusion of SDD in standard SRP therapy leads to a reduction in pocket depth by 0.8 mm more than with SRP alone. In addition, it has been demonstrated that a 3-month course of SDD leads to further improvement in clinical periodontal parameters and markers of periodontal and bone tissue destruction (C-telopeptides, tartrate-resistant acid phosphatase, and osteoprotegerin) in the gingival crevicular fluid over a 12-month period, without causing significant changes in the subgingival microbiota or increasing antibiotic resistance. The effect of SDD is also associated with the downregulation of myeloperoxidase expression and a reduction in oxidative stress. Currently, in international periodontics practice, SDD (20 mg twice a day, taken 1 h before or 2 h after meals for a duration of 3 months to 2 years, depending on the severity of bone loss) is used in conjunction with traditional non-surgical methods such as SRP. In addition, dental formulations of doxycycline for subgingival administration are utilized, including dental gels and non-resorbable dental fibers made of synthetic polymers (295, 296). In addition to antibiotics and host modulators, antioxidants and bioregulatory agents play an important role—coenzyme Q10, resveratrol, green tea polyphenols, and omega-3 fatty acids. They reduce oxidative stress, suppress the production of proinflammatory cytokines, and improve tissue metabolism. It has been shown that taking omega-3 fatty acids at a dose of 2 g/day for 3 months reduced the bleeding rate by 35% and contributed to a faster recovery of clinical attachment (297–299).

Furthermore, within the framework of HMT, the use of the antioxidant CMC2.24—a novel 4-(phenylaminocarbonyl)-chemically modified curcumin—is experimentally justified. CMC2.24 acts as a pleiotropic matrix metalloproteinase inhibitor; it has been shown to reduce the activation of pathologically active MMP-9, normalize diabetic osteoporosis, and promote the resolution of inflammation, without exerting any effect on hyperglycemia in diabetic rats (300). Furthermore, within the framework of HMT, sodium selenite (Selenase), a selenium formulation, is of substantial interest. When administered as a course in rats with experimental chronic periodontitis, it reduces periodontal pocket depth alongside a decrease in blood levels of the inflammatory markers TNF-α and IL-1β, as well as the oxidative stress marker nitrotyrosine. The adjunctive inclusion of Selenase in the comprehensive treatment of patients with CGP exerts a potentiation of anti-inflammatory and reparative effects. At 30 days post-treatment, a statistically significant positive trend is observed, characterized by a reduction in the papillary–marginal–alveolar (PMA) index, papillary bleeding index (PBI), PPD, MMP-2, TNF-α, and IL-1β (301, 302). It can be hypothesized that a promising direction for HMT could be the pharmacological activation of HSP70/GSH endogenous cytoprotection mechanisms aimed at suppressing inflammation, inhibiting oxidative stress, normalizing the nitroxidergic system, reducing focal ischemia, and improving the overall condition of periodontal tissues. In cases where conservative therapy and adjuvant methods do not lead to a satisfactory state of tissues, the logical step is the use of surgical and regenerative technologies. Surgical interventions are aimed at opening access to deep periodontal pockets, removing granulation tissues, smoothing root surfaces, and forming a shape favorable for further regeneration. The classic ones include flap operations, curettage, osteoplasty, and gingivectomy, which allow eliminating granulation tissues, reducing the depth of pockets, and restoring the architecture of the gums. A number of studies have shown that open flap surgery provides a significantly greater reduction in the depth of probing compared with closed curettage (303, 304). Surgical treatment provides an average reduction in pocket depth of 0.5–0.8 mm more than non-surgical treatment for defects greater than 6 mm. However, surgical intervention is indicated only after resolution of inflammation and with adequate hygiene. Indications for surgical treatment include persistence of periodontal pockets ≥5–6 mm deep after non-surgical therapy; presence of intraosseous and furcation defects; pathological changes in the contours of the bone and mucosa (305, 306).

A classic example is the Modified Widman Flap, described by Widman (1918) and improved by Goldman and Cohen (1958). It involves making an internal oblique incision, exfoliating the muco-apical flap, carefully removing granulations and tartar, root planing, and repositioning the flap at the original level (307). Flap operations can achieve an improvement in attachment by 1.5–2 mm in the case of exit pockets >6 mm. In the case of pronounced deformations of the bone edges, an apically displaced flap with osteoplasty is used, which improves access and control, but is accompanied by greater gingival recession (308). The latest approaches include microsurgical techniques using optics and atraumatic instruments, which contribute to faster healing and less blood loss. In recent years, regenerative technologies aimed at restoring lost periodontal tissues have gained popularity. The regenerative approach is key in modern therapy. The guided tissue regeneration technique involves the use of barrier membranes that prevent the migration of epithelial cells into the defect, providing space for the proliferation of connective tissue cells and osteoblasts. When combining membranes with bone substitutes or autogenous bone, a better effect is observed: bone growth is 3.5–4.2 mm in vertical defects within 12 months (309, 310).

Nanocomposite hydroxyapatite materials with controlled release of growth factors have also been used, which have shown bone growth of up to 3 mm in 9 months. When defects occupy large spaces or have complex morphology, the use of 3D-printed bioskeletons and bioactive polymers is useful. The introduction of 3D bioprinting technology allows the creation of personalized biotemplates that precisely match the shape of the patient's bone defect. These structures can be individually adapted to the anatomy of the patient's defect, saturated with cells and growth factors, ensuring bone and tissue regeneration (311, 312). The development of individual regenerative constructs using bioactive polymers and 3D printing is one of the most promising areas in the future treatment of chronic kidney disease. Current innovations focus on biomaterials with nanostructured components, collagen matrices with nanoparticles, hydrogels with functional peptides, and synthetic polymers with a minor antibacterial effect (313, 314). Such preparations have the ability to sequentially release growth factors and stimulate cell migration and angiogenesis in defects, which increases the prognostic efficiency of regeneration. Another promising technology is the use of growth factors (PDGF, BMP, TGF-β, and VEGF), which activate cell proliferation and angiogenesis. The introduction of autologous platelet-rich plasma (PRP and PRF) promotes the formation of a fibrin matrix and faster osteoregeneration (315, 316).

One of the promising technologies is tissue engineering and cellular methods—transplantation of mesenchymal stem cells (MSCs), bioactive scaffolds with stem cells, and bioreactor models. Tissue engineering based on the use of mesenchymal stem cells opens up new prospects for the restoration of periodontal tissues. The source of MSCs can be bone marrow, umbilical cord blood, adipose tissue, and periodontal ligaments. These cells have the potential to differentiate into osteoblasts, cementocytes, and fibroblasts. In experimental models, the use of MSCs on biocompatible matrices leads to a full restoration of the periodontal ligament and alveolar bone (317, 318). Recent clinical studies have shown that the use of autologous stem cells, in combination with hydroxyapatite, increases bone density by 25% and reduces healing time after surgery by 30% (319, 320). Laser techniques in the regenerative context complement surgery. Lasers can sterilize pockets, coagulate vessels, and reduce bleeding. Laser irradiation after membrane implantation can stimulate angiogenesis and accelerate material integration. Meta-analyses show that combining SRP with laser irradiation provides an additional 0.4–0.6 mm improvement in clinical attachment (321). Despite these successes, there are limitations. Some regenerative methods are expensive, technologically difficult to use, require skilled surgeons, and carry the risk of unpredictable material resorption. All innovations require long-term monitoring, standardization of protocols, and randomized controlled trials (322). The final stage of comprehensive treatment is SPT, aimed at preventing relapse. As part of the analytical approach to assessing the effectiveness of therapy, the use of meta-analyses and systematic reviews is important. The patient should visit the dentist every 3–6 months for professional cleaning, hygiene control, and assessment of tissue condition. Patients with regular SPT visits have a 45% lower risk of tooth loss over a period 10 years. The absence of SPT increases the risk of relapse by 5 times (323, 324). Thus, modern approaches to the treatment of periodontitis combine traditional methods with progressive technologies. A comparison of international and national treatment protocols shows that the development trends in the treatment of chronic generalized periodontitis are focused on a biointegrative and personalized approach. Classical methods (SRP, surgery) remain the basis, but they are enhanced by modern technologies—regenerative biomaterials, host modulators, antioxidants, laser therapy, and photodynamic therapy.

## 6 Cytokine modulators in the treatment of periodontitis

### 6.1 General pharmacological characteristics of cytokine modulators

Over the past decade, the emergence of novel therapeutic classes has fundamentally transformed the clinical management of various autoimmune, allergic, infectious, and chronic inflammatory disorders. These drugs are divided into three groups: cytokines, monoclonal antibodies, and fusion proteins. Biological drugs have fewer side effects than traditional medications and can act on specific target cells (325–337).

An analysis of the use of recombinant cytokine analogues in patients revealed a dependence of clinical efficacy and the severity of adverse reactions on individual patient characteristics: the nature of the inflammatory response depended on the functional polymorphism of cytokine genes. The results obtained suggest that for carriers of highly producing IL-1RN gene variants, additional administration of their recombinant analogues will not have significant results and may be accompanied by the development of adverse reactions, and in some cases, activate the pathological process. Similarly, in carriers of highly producing IL-1RN*2 gene variants, the effect of administration of recombinant analogues of IL-1RА or other anti-inflammatory cytokines will be less pronounced than in patients with a “pro-inflammatory” genotype. Clinical trials of antibodies to TNF-α have been successful. In 1992, in an open trial, it was possible to demonstrate for the first time the positive effect of bizarre human antibodies specific to TNF-α (infliximab) in 20 patients with rheumatoid arthritis. The drug contributed to the reversal of the clinical symptoms of the disease, significantly reduced the level of CRP in the blood plasma, and decreased the erythrocyte sedimentation rate. These results were confirmed in large-scale placebo-controlled clinical trials in which infliximab was used in combination with methotrexate, which gave the FDA grounds to recommend infliximab for the treatment of patients with rheumatoid arthritis. Subsequently, along with infliximab, the FDA approved the clinical use of several other drugs, including etanercept, which inhibits the binding of TNF-α and TNF-β to specific membrane receptors, and adalimumab, which promotes the inactivation of TNF-α and subsequent blocking of interaction with the cell surface receptors of TNF p55 and p75. In addition, infliximab and adalimumab, in combination with methotrexate, contributed to a slight increase in the incidence of serious infectious complications. The use of more stringent ACR criteria revealed some advantages of etanercept (328–330). However, the results of many controlled trials show that a significant proportion of patients with rheumatoid arthritis do not respond to treatment with TNF-α antagonists. Induced tolerance to biologically active drugs often occurs during therapy, although there are also contrary observations. All of this dictates the urgent need to find new targets for drug-induced correction of TNF-α effects (331, 332). The results of clinical trials using tocilizumab, a drug that contains human monoclonal antibodies specific to IL-6 receptors, have shown high efficacy of tocilizumab in reversing disease activity and preventing the formation of destructive changes in cases of rheumatoid arthritis resistance to traditional DMARDs (disease-modifying antirheumatic drugs) (333). At the same time, the potential toxic effects of this treatment strategy have not yet been clearly defined; it is assumed that the drug may have some limitations in terms of cardiovascular safety. The therapeutic effect of an IL-1 receptor antagonist in experimental cerebral stroke has also been studied. In the pathogenesis of acute cerebrovascular accidents, various links in the neurodestructive cascade play a key role; they are interconnected and time-determined. At the site of hypoxia/ischemia, endothelial cells, leukocytes, and macrophages are activated, producing cytokines such as interleukin (334). A “cytokine cascade” then develops—overproduction of proinflammatory cytokines and a relative deficiency of anti-inflammatory cytokines and growth factors. Initially, production of IL-1 increases, which is the main mediator of the local inflammatory reaction and the acute-phase response at the organ level. This cascade coordinates the “cytokine cascade”—the ratio of pro- and anti-inflammatory mediators that induce and maintain inflammation at the site of hypoxia/ischemia, leading to changes in microcirculation, the blood–brain barrier, and delayed neuronal death (335). IL-1 expression induces the synthesis of IL-1RА, which inhibits the action of IL-1 by competitively binding its specific membrane type I receptors and prevents the interaction of the IL-1 receptor with the acceptor (accessory) protein, leading to a complete absence of signal transmission into the cell. Therefore, the use of interleukin-based cytokine preparations is an important promising link in the effective protection of brain tissue in the complex therapy of cerebrovascular diseases (336, 337). The influence of recombinant IL-1 on the dynamics of posthypoxic changes in the brain tissues of rats with experimental focal stroke, namely, the functional activity of mitochondria and the thiol-disulfide system, was established (338).

#### 6.1.1 TNF-α inhibitors

TNF-α inhibitors, which include etanercept, infliximab, adalimumab, certolizumab pegol, and golimumab, are FDA-sanctioned biologic therapies used to treat rheumatoid arthritis, Crohn's disease, ankylosing spondylitis, ulcerative colitis, plaque psoriasis, uveitis, and hidradenitis suppurativa (329, 339, 340). There are also a number of off-label indications for dental use. Experimental studies have shown that TNF-α blockade reduces periodontal inflammation in models of chronic periodontitis. For example, researchers found that administration of etanercept (a human dimeric fusion protein that blocks TNF-α) to rats with ligature-induced chronic periodontitis and prooxidant-induced periodontitis reduced elevated neutrophil levels, decreased the severity of periodontal inflammation, and reduced local bone damage. Infliximab is a monoclonal antibody against TNF-α. It reduces IL-1β expression in the gums and exerts significant anti-inflammatory and bone-protective effects in Wistar rats with experimental periodontitis. Clinical studies using TNF blockers such as infliximab (a chimeric antibody against human TNF) have suggested the clinical significance of TNF in the control of periodontal parameters (Table 2) (341–343).

**Table 2:** Pharmacotherapeutic characteristics of cytokine receptor modulators in periodontitis.

Drug, mechanism of action	Pharmacodynamics	Preclinical studies	Clinical use results	Adverse reactions
Anakinra, an IL-1 blocker (384–387)	A modified interleukin-1β receptor antagonist. Competitive inhibition of IL-1α and IL-1β binding to the specific type I receptor (IL-1RI), resulting in a reduction in the intensity of proinflammatory activation, reversal of symptoms, and limitation of inflammation progression	*In vitro* cell cultures demonstrate that Anakinra suppresses IL-1β-mediated leukocyte clustering and osteoclastogenesis. Furthermore, topically applying a 1% Anakinra dental gel (1 mg/kg) results in a reduction of gingival pocket depth down to 2.5 mm, accompanied by a virtually complete resolution of clinical inflammation, swelling, and bleeding. In the blood of experimental animals, this therapeutic intervention induces specific biochemical changes, including a decrease in iNOS expression and nitrotyrosine concentration, along with a concomitant increase in reduced glutathione levels, glutathione peroxidase-4 (GPx4) expression, and Cu/ZnSOD. In addition, treatment with the dental gel lowers the expression of systemic inflammatory markers, namely, TNF-α, MMP-2, and IL-1β, while simultaneously upregulating the concentrations of cytoprotective proteins HIF-1 and HSP70	Additional inclusion of Anakinra in 60 patients with periodontitis (Stage II). (1 mg/day, 5 sessions of electrophoresis) leads to a decrease in MMP-2 and nitrotyrosine, increases the activity of glutathione reductase and glutathione peroxidase, a decrease in LDH, a decrease in iNOS expression and an increase in eNOS, a decrease in bleeding indices, bone tissue destruction indices on day 90 compared with similar laboratory and clinical parameters of patients recorded before the start of combination therapy including anakinra	Local reactions at the injection site (pain, redness, swelling), headaches, infections (respiratory, sinusitis), and increased cholesterol levels. No adverse reactions were observed during electrophoresis in patients with periodontitis (Stage II)
Tocilizumab, an IL-6 blocker (388–390)	By inhibiting soluble and membrane IL-6 receptors, it interrupts the signaling pathway that causes inflammation, rapidly reducing CRP, matrix metalloproteinases, and the erythrocyte sedimentation rate (ESR)	*In vitro* (17,353 cases), decreased IL-6 signaling activity was associated with a lower risk of periodontitis (odds ratio (OR) = 0.81 per 1 unit decrease in log CRP level)	Short-term use of tocilizumab (60 patients with periodontitis) significantly reduced gingival and periodontal inflammation, as evidenced by a reduction in the gingival inflammation index, bleeding on probing, and periodontal pocket depth, as well as decreased levels of TNF-α, total Immunoglobulin G, and serum amyloid	Increased risk of infections (including tuberculosis), liver dysfunction, and gastrointestinal perforations. Headache and increased blood pressure
Rilonacept, a high-affinity IL-1β blocker (345, 391, 392)	Soluble decoy receptor. It binds circulating IL-1α and IL-1β, inhibiting their activity. It has a high affinity for IL-1β and a lower affinity for IL-1β. It reduces systemic inflammation, as evidenced by a rapid decrease in C-reactive protein levels	No studies have been conducted; reviews contain theoretical assumptions regarding its use in the treatment of periodontitis	Not researched	Increased liver enzyme levels, abdominal pain, upper respiratory tract infections, cough, and headache. Neutropenia
Canakinumab, a specific IL-1β blocker (391)	A monoclonal antibody specific to interleukin-1β. It blocks IL-1β-induced gene activation and the production of inflammatory mediators such as IL-6 and COX-2	No separate studies have been conducted; reviews contain theoretical assumptions regarding its use in the treatment of periodontitis	Not researched	Decreased immunity, risk of cancer, rhabdomyolysi s, and osteonecrosis
Etanercept, a TNF-α blocker (334, 335, 393, 394)	It competitively inhibits the binding of TNF-α and TNF-β (lymphotoxin-α) to TNF receptors (p55 and p75) on the cell surface and blocks the inflammatory response cascade, leading to a decrease in IL-6, adhesion molecules, and matrix metalloproteinases (MMP-1 and MMP-3)	Reduces elevated levels of neutrophils, iNOS, IL-6, and nitrotyrosine, reduces the severity of periodontal inflammation, and reduces local bone damage in experimental periodontitis	Etanercept did not demonstrate a significant improvement in periodontal health in patients. However, a significant improvement in the Korach Dental Anxiety Scale score was noted, as well as a significant reduction in C-reactive protein and ESR levels	Infusion site reactions (itching, redness), infections (including tuberculosis), headache, and nausea
Infliximab, a TNF-α blocker (334–338, 394)	This chimeric IgG1 kappa monoclonal antibody acts as a high-affinity blocker of TNF-α receptors (TNF-αA and TNF-αB) and disrupts proinflammatory cascades, reduces leukocyte migration, induces the apoptosis of activated T cells, and reduces levels of inflammatory markers in blood serum, such as IL-1 and IL-6	Reduces the number of granulocytes in the blood, IL-1β, TNF-α, and myeloperoxidase levels in the gums and has a significant anti-inflammatory and bone-protective effect in rats with experimental periodontitis	With infliximab, a significant reduction in CAL, BOP, and probing depth (PD) was observed. There was also a reduction in MMP-3 and TNF-α levels in the patients’ gingival fluid. However, another study found no significant changes in PD and BOP	Infusion reactions, infections, headache, stomach pain, nausea, and fatigue. Serious risks include severe infections (tuberculosis, pneumonia), lymphoma, heart failure, and liver damage

For instance, patients with rheumatoid arthritis undergoing anti-TNF-α regimens display significantly improved periodontal indices, including plaque and gingival scores, probing pocket depth, clinical attachment loss, and bleeding on probing, coinciding with reduced TNF-α concentrations within the gingival crevicular fluid. Furthermore, longitudinal evaluations of rheumatoid arthritis cohorts receiving the fully humanized monoclonal antibody adalimumab confirm these therapeutic benefits. Three months postinitiation, anti-TNF-α therapy yielded marked reductions in the gingival index, bleeding scores, and probing depths, changes that correlated tightly to a systemic downregulation of serum TNF-α and IL-6 levels (344–346). Despite these clinical advantages, TNF-α inhibitors are tied to severe adverse events, including drug-induced lupus erythematosus, congestive heart failure, severe infections, sepsis, and an increased risk of malignancies (329). TNF inhibitors also exhibit unexplained side effects, including impacts on oral bone tissue. For example, the administration of adalimumab and infliximab resulted in the development of osteonecrosis of the jaw after oral and intravenous bisphosphonate therapy and impaired oral healing (339, 347, 348).

The above limits their use in periodontology. However, in periodontitis, the pharmacological blockade of IL-1β possesses key advantages over TNF-α blockade, as IL-1β acts as a highly potent local factor that stimulates bone resorption and tissue destruction in the periodontium. Therefore, the pharmacological blockade of IL-1β directly targets specific osteoclast formation and extracellular matrix degradation, without exerting a significant impact on systemic immunity (164, 349).

#### 6.1.2 IL-6 blockers

*In vivo* blockade of IL-6-dependent STAT3 activation has been shown to inhibit periapical bone resorption and attenuate the apical infiltration of immune cell populations such as macrophages, thereby underscoring the therapeutic potential of targeting the IL-6/STAT3 signaling axis (216). Consistently, systemic administration of tocilizumab—a humanized anti-IL-6R monoclonal antibody—in experimental models of ligature-induced periodontitis effectively suppresses inflammatory cell recruitment, downregulates Th17-associated cytokines, and attenuates pathological RANKL expression. Furthermore, this therapeutic intervention significantly minimizes alveolar bone resorption and clinical attachment loss, indicating that targeted IL-6R blockade represents a highly promising approach for the management of periodontitis (214, 350).

#### 6.1.3 IL-1β blockers

The IL-1 family represents the primary cytokine cohort responsible for the initiation and maintenance of periodontal inflammation. Within the inflamed microenvironment, IL-1β plays a key role in augmenting localized blood flow, driving leukocyte recruitment, and promoting neutrophil infiltration. Furthermore, IL-1β upregulates MMP-9 expression across diverse cell lineages involved in periodontal pathology, including osteoblasts, osteoclasts, neutrophils, and cementoblasts (342). It concurrently stimulates the production of other matrix metalloproteinases such as MMP-1 and MMP-3 in human periodontal ligament and gingival fibroblast cells, thereby accelerating extracellular matrix degradation, alveolar bone resorption, and structural tissue breakdown. Given these central mechanisms, members of the IL-1 family have emerged as promising therapeutic targets for the management of oral inflammatory disorders (351, 352). However, the clinical efficacy of IL-1 inhibitors possessing varying selectivities and affinities remains only partially characterized in preclinical and clinical settings. Although conventional antimicrobial agents—including minocycline, doxycycline, roxithromycin, amoxicillin, and metronidazole deployed topically within periodontal pockets as microspheres—improve objective clinical parameters, their direct impact on local IL-1β concentrations is highly constrained (353, 354), with these therapeutic effects typically diminishing after a 6-month follow-up period (281). Consequently, engineered IL-1β antibodies and specific receptor antagonists represent a subject of intense interest for both pharmacologists and clinicians (355).

**Rilonacept** is a recombinant IL-1β antagonist comprising the extracellular domains of both the human IL-1β receptor and the IL-1β receptor accessory protein linked to the Fc portion of human IgG1, thereby operating as an uncoupling “IL-1 decoy” (Table 2) (356, 357). In 2008, it secured FDA approval for the management of cryopyrin-associated periodic syndromes (CAPS). Currently, a series of clinical trials are investigating the therapeutic utility of rilonacept across several chronic inflammatory pathologies, including type 1 diabetes, atherosclerosis, chronic kidney disease, and hepatitis (NCT01903798) (358, 359). To date, however, evaluations exploring its clinical deployment in inflammatory disorders of the oral cavity remain nonexistent.

Canakinumab is a fully human monoclonal antibody with high affinity designed to specifically target, bind, and neutralize human IL-1β. The FDA approved canakinumab in 2009 for managing chronic obstructive pulmonary disease, osteoarthritis, type II diabetes, rheumatoid arthritis, and atherosclerosis. This agent represents the third IL-1 inhibitor approved for treating autoimmune disorders and the second medication approved for managing CAPS. Canakinumab offers distinct therapeutic benefits compared with alternative IL-1 antagonists. In contrast to anakinra and rilonacept, which interrupt signaling cascades for both the IL-1*α* and IL-1β isoforms, canakinumab exhibits strict specificity toward IL-1β, leaving other IL-1-mediated pathways unaffected. Furthermore, because of its prolonged half-life, canakinumab achieves sustained inhibition of IL-1β over an extended time frame (360–362). However, canakinumab suppresses immune function, can elevate cancer risks, and has been associated with rhabdomyolysis and osteonecrosis The identified side effects have diminished research interest in canakinumab for its potential use in periodontology.

Natural plant-derived IL-1 inhibitors, particularly the polyphenolic compounds resveratrol and curcumin, are also being considered as viable anticytokine agents. *In vivo* experimental models of periodontitis show that these compounds can reduce local IL-1β expression and limit alveolar bone loss. Preclinical data emphasize that curcumin is effective in treating periodontitis because of its diverse molecular targets. Curcumin reduces inflammation in periodontal tissues by inhibiting several proinflammatory cytokines and enzymes, including IL-1β, TNF-α, MMPs, prostaglandin E2 (PGE2), and cyclooxygenase-2 (COX-2). Concurrently, it increases the presence of the anti-inflammatory cytokines IL-4 and IL-10. Furthermore, curcumin inhibits key transcription factors involved in the immune response, such as nuclear factor-kappa B (NF-κB) and signal transducer and activator of transcription 1. Finally, administration of this polyphenol lowers additional biomarkers associated with periodontitis severity, such as CRP, alkaline phosphatase, and procalcitonin (363–370).

The anti-inflammatory effects of curcumin are comparable to the combination of chlorhexidine and metronidazole. *In vitro* studies demonstrate that curcumin diminishes the production of IL-1β and TNF-α in rat gingival fibroblasts stimulated by LPS (371, 372).

Resveratrol reduces the levels of proinflammatory cytokines such as IL-1β and TNF-α in periodontitis by inhibiting the NF-κB and MAPK signaling pathways activated by LPS. By suppressing these key pathways, resveratrol helps protect periodontal tissues from damage caused by chronic inflammation. Resveratrol blocks these signaling cascades, particularly the NF-κB and MAPK pathways, preventing the subsequent production of inflammatory molecules (373, 374). A hyaluronic hydrogel reinforced with solid lipid nanoparticles containing resveratrol was developed. Treatment with this gel resulted in a decrease in IL-1β and TNF-α, suppressed the formation of ROS and lipid peroxidation products, improved mitochondrial function, and enhanced osteoblast differentiation.

Experimental evidence substantiates the IL-1β inhibitory properties of metformin (375). Mechanistically, metformin activates AMP-activated protein kinase (AMPK), an enzyme documented to exert prominent anti-inflammatory and immunomodulatory effects. In rat models of experimental periodontitis, metformin administration reduces local IL-1β expression and mitigates alveolar bone resorption. Morphometric and histological evaluations through microcomputed tomography confirm that metformin treatment promotes the structural restoration of the periodontal ligament, alveolar bone, and cementum damaged by periodontitis. This therapeutic outcome is closely tied to a reduction in persistent inflammatory activity during the resolution phase of periodontitis, which is typically hindered by sustained IL-1β levels that arrest tissue regeneration. *In vitro* models simulating these pathological conditions demonstrate that AMPK activation by metformin antagonizes IL-1β signaling, inhibits osteoclast differentiation, and restores the osteogenic differentiation capacity of cementoblasts and periodontal ligament cells (376, 377). Concurrently, interest has centered on RAIL, a selective recombinant IL-1β antagonist biotechnologically synthesized using an Escherichia coli TG1 (pTAC-hIL-1ra) expression system. This agent is a 153-amino acid peptide with a molecular weight of 17.9 kDa. RAIL effectively interrupts the IL-1β-dependent cascades that drive ischemic neurodestruction. Accumulating data indicate that RAIL normalizes GSH-dependent pathways governing HSP70 expression within both the cytosol and mitochondria during acute cerebral ischemia. Recently, an intranasal RAIL gel formulation (5 mg/mL) was engineered at the Department of Drug Technology of Zaporizhzhia State Medical and Pharmaceutical University. Preclinical evaluations have demonstrated that this RAIL gel exhibits exceptional neuroprotective, antioxidant, anti-ischemic, and antiapoptotic properties alongside a favorable safety profile (378–380).

Certain antibodies or antagonists also exert an indirect influence on IL-1β. For example, the proteasome inhibitor bortezomib, which is utilized as an anticancer drug, suppresses IL-1β expression and prevents alveolar bone loss in experimental periodontitis models (381). Collectively, these findings indicate that employing IL-1β blockers represents a promising strategic approach for periodontitis therapy. Anakinra, a recombinant homolog of the human IL-1 receptor antagonist, was initially approved in the United States and Europe in 2001 to treat rheumatoid arthritis, and it has since demonstrated efficacy across various inflammatory conditions. As the first approved therapeutic agent targeting IL-1 signaling, it is a non-glycosylated human recombinant IL-1RA that binds to the IL-1 receptor 1, competitively blocking both IL-1*α* and IL-1β from binding (382). Multiple studies show that hindering the effects of IL-1β in rheumatoid arthritis protects bone and cartilage architectures (383). *In vitro* research on cartilage tissue revealed that IL-1RA reduced the destruction caused by synovial fibroblasts by up to 45%. Similarly, *in vitro* evaluations on bone sections showed that IL-1RA lowered the rate of bone resorption and blocked the development of osteoclast-like cells when cultured alongside IL-1β (384). Animal studies have confirmed that IL-1RA decreases cartilage degradation and bone erosion. Furthermore, IL-1RA knockout mice display an increased count of osteoclast precursors, particularly within the long bones and jaw. Depending on the specific type of precursor, IL-1β acts as an osteoclast activator to alter osteoclast functional activity and lifespan (385).

Clinical trials have also demonstrated that IL-1RA slows the radiographic progression of rheumatoid arthritis, likely by preventing the stimulatory effects of IL-1 on osteoclasts. Consequently, anakinra is established as a safe and effective option for treating rheumatoid arthritis (384, 386). Because IL-1*α* triggers osteoblast apoptosis and suppresses osteoblast differentiation, it is anticipated that IL-1RA would counter these negative impacts, thereby increasing osteoblast activity. Given the clear pathological parallels between rheumatoid arthritis and periodontitis, it is noteworthy that anakinra—a potent inhibitor of their common denominator, IL-1β—has not been widely investigated in periodontitis models. These encouraging results from clinical, *in vitro*, and *in vivo* investigations support the value of further evaluation using a suitable cell system. Periodontal ligament fibroblasts (PDLFs) are connective tissue cells that anchor teeth to the bone and play an essential role in both osteoclastogenesis and osteogenesis. A study demonstrated that anakinra exerted an inhibitory effect on osteoclastogenesis in both the presence and absence of IL-1β within PDLF cultures (387). These factors combined make investigating the therapeutic efficacy of anakinra in periodontitis a highly promising and valuable direction. Accordingly, we developed an anti-inflammatory oromucosal gel formulation containing anakinra designed for the prevention and comprehensive treatment of inflammatory periodontal diseases. Investigations established that the consistency and mucoadhesive properties of this anakinra-loaded oromucosal gel are significantly enhanced by incorporating Na CMC and Tween-80 into the formulation. The resulting oromucosal gel shows satisfactory kinetic stability and thixotropic properties. This developed dental gel containing anakinra fulfills all safety and security criteria required for dosage forms in this category, showing low toxicity and an absence of local irritation or allergenic reactions (388, 389).

Therapeutic administration of a dental gel containing the IL-1 receptor antagonist anakinra (1 mg/kg) to animals with periodontitis induced by a calcium-deficient, pro-oxidant model significantly improved clinical outcomes, reducing probing pocket depth from 8 mm to 2.2 mm and virtually eliminating local hemorrhage and edema. This phenotypic resolution was accompanied by a 37.8% downregulation of iNOS expression (*p* < 0.05), alongside reductions in circulating nitrotyrosine and total nitric oxide metabolites (NOx) by 55.2% and 30%, respectively (*p* < 0.05). Furthermore, this intervention expanded the antioxidant defense system, elevating reduced glutathione levels by 63% (*p* < 0.05), glutathione peroxidase-4 (GPx4) expression by 60.4% (*p* < 0.05), and Cu/Zn-SOD by 31.2% (*p* < 0.05). Concurrently, systemic inflammatory markers were suppressed relative to untreated cohorts, with TNF-α decreasing by 82% (*p* < 0.05), MMP-2 by 65% (*p* < 0.05), and IL-1β by 71.4% (*p* < 0.05). Local anakinra delivery also augmented survival and adaptation markers, driving a 42% upregulation of HIF-1*α* (*p* < 0.05) alongside a 62.8% increase in HSP70 relative to the control group, representing a 2.4-fold elevation (*p* < 0.05) over intact baselines (390, 391).

Translating these findings into a clinical setting, incorporating anakinra (1 mg/day) into the comprehensive rehabilitation matrix of 60 patients who presented with periodontitis (Stage II) yielded markedly superior therapeutic outcomes compared with standard clinical regimens. These advancements were corroborated by clinical examinations, biochemical assays, and ELISA mapping, which collectively indicated a distinct reduction in active markers governing inflammation, oxidative stress, ischemia, and endothelial nitric oxide dysregulation. Ultimately, adjunctive anakinra therapy potentiated the anti-inflammatory efficacy of standard treatments, accelerating the reduction of local MMP-2 levels and stabilizing clinical parameters such as pocket depth, bleeding, and tooth mobility. It likewise reinforced the antioxidant arm of combination therapy—manifested by enhanced glutathione reductase and glutathione peroxidase activities (*p* < 0.05) and lower nitrotyrosine levels—while amplifying anti-ischemic protection through reduced LDH and increased succinate dehydrogenase concentrations (*p* < 0.05). Consequently, the incorporation of anakinra effectively suppressed pathological iNOS expression and contributed to a compensatory increase in homeostatic endothelial nitric oxide synthase (eNOS) signaling compared with similar parameters in patients in the control group (390).

## 7 Clinical and translational implications

The recognition of the key role of cytokines in the pathogenesis of periodontal diseases, acting as mediators of inflammation, namely, in the initiation of the destruction of connective tissue and the bone structure of the tooth, opens up significant opportunities for translational medicine in the field of identification of biomarkers, therapeutic strategies, and the search for new drug targets. Monitoring their level in oral fluid (saliva), gingival fluid, or blood is a highly sensitive method for early diagnosis of gingivitis and periodontitis, as well as a negative prognosis for the formation of mechanisms of damage to target organs such as the heart, brain, liver, kidneys, and pancreas.

The main cytokine markers of periodontal damage have a high informative potential: IL-1: оne of the main markers of the activity of the inflammatory process, stimulates bone resorption and collagen destruction. Periodontitis can increase systemic levels of proinflammatory cytokines and thus can provoke or intensify inflammatory, degenerative, or neoplastic processes in the body. Thus, IL-1β can be not only a key, highly sensitive marker of the prognosis, activity, and severity of periodontitis, but also an informative marker of the prognosis of a high risk of developing related systemic diseases such as diabetes, cardiovascular diseases, and rheumatoid arthritis; TNF-α: proinflammatory cytokine, which sharply increases in gingivitis and periodontitis, contributing to tissue damage; IL-6: mediator of the acute phase of inflammation, takes part in the destruction of the periodontium; IL-10: a key anti-inflammatory cytokine that plays an important role in the immunological mechanisms of periodontal diseases. Its level and balance with proinflammatory cytokines (such as IL-1β and TNF*α*) serves as a marker of severity, activity of inflammation, and progression of tissue destruction. The content of IL-10 in saliva and periodontal fluid correlates with the clinical indicators of inflammation, such as the depth of periodontal pockets and bleeding gums; IL-17 and IL-23: included in the pathogenesis of tissue destruction; IFN-γ: an important proinflammatory cytokine and a marker of periodontal tissue damage that indicates the activation of a cellular immune response (Th1-type), its increased concentration in oral fluid (saliva) and gum fluid points to the inflammatory process and activity of chronic generalized periodontitis and the progressive destruction of bone tissue and loss of tooth attachment (185, 186, 193).

An analysis of the cytokine profile allows not only to diagnose the disease in the early stages, but also to assess the effectiveness of the anti-inflammatory therapy, because a decrease in the level of these markers indicates the subsidence of the pathological process. IL-1β activates the expression of matrix MMР in human periodontal ligament cells and gingival fibroblast cells, which contribute to the degradation of the extracellular matrix and, in turn, lead to bone resorption and tissue destruction. Thеrеfore, MMР-2 can be used as a secondary informative marker of periodontitis, an increase in the concentration of which in the oral fluid is reliably associated with the severity of periodontitis. IL-1β activates signaling pathways (e.g., NF-κB and MAPK) against the background of glutathione depletion, leading to the damage of lipids, proteins, and DNA inside cells, which, in turn, leads to structural and functional disorders in periodontal tissues and accelerates the progression of the disease. Oxidative stress also contributes to the continuity and severity of inflammatory reactions, thereby accelerating pathological tissue damage and aggravating the development of periodontal diseases. Therefore, stable metabolites of oxidative stress—nitrotyrosine, 8-hydroxyguanine, and MDA, as well as the level of glutathione, and the activity of glutathione reductase and glutathioperoxidase can be used as secondary informative markers for assessing the severity of periodontitis and the effectiveness of therapy. All this justifies the use of IL-1 modulators in therapeutic dentistry. The most encouraging results are revealed when using the IL-1 receptor antagonist anakinra. It is shown that the pharmacological blockade of IL-1β in periodontitis leads to the activation of endogenous cytoprotection, then to normalization of the NO system and the glutathione system, and finally to a reduction of oxidative stress, ischemia, and clinical symptoms of the disease (384, 385).

For the first time, the therapeutic potential of a new strategy of pharmacological blockade of IL-1β, which may have new prospects in the treatment of chronic generalized periodontitis, has been demonstrated. The inclusion in the complex therapy of anakinra (1.0 μg/day, with the help of electrophoresis) significantly increases the effectiveness of the basic therapy—it potentiates its anti-inflammatory effect and also leads to the appearance of an antioxidant effect, as well as an anti-ischemic effect, as well as to an improvement in the condition of the periodontium, a decrease in bleeding, and tooth mobility for 90 days, both in relation to the data obtained before treatment and to the values of the indicators of the control group. A new promising medicinal form of anakinra—dental gel—has been developed for clinical use in dentistry. The expediency of the wide clinical application of anakinra, both in view of new medicinal forms and in view of electrophoresis in the complex treatment of periodontitis of moderate severity, is shown (384). Antioxidants, in particular, selenium preparations, can also be used to enhance the action of cytokine receptor blockers (301, 302). The pharmacological blockade of IL-1β with various drugs, including anakinra in various dosage forms and routes of administration, appears to be more rational and promising in the complex therapy of periodontitis than the use of other traditional drugs such as doxycycline in a sub-antimicrobial dose (to inhibit matrix metalloproteins, reduce inflammation, and tissue destruction) and metronidazole (to reduce inflammation, bleeding, and periodontal pockets). Thus, the effect of doxycycline and metronidazole on IL-1β is limited. The effect disappears during subsequent observation for 6 months. This is confirmed by preclinical and clinical observations (335, 387). This is primarily due to the more directed, targeted action of IL-1β blockers, which leads to the interruption of inflammatory, apoptotic, and oxidative stress signaling pathways at sites more sensitive to pharmacological intervention, in contrast to SDD, which acts on less promising sites of the signaling pathways. Thus, in the groups of patients with periodontitis, the administration of anakinra as part of standard complex therapy (intraoral bimaxillary transgingival electrophoresis, 1 mg/day) (30 patients) provides a more pronounced improvement in clinical indicators compared with standard complex therapy combined with the use of SDD (30 patients). This is manifested after 90 days by a more pronounced, significant reduction in PPD, PBI, HI (OHI-S) (oral hygiene index simplified), and PMA index, indicating a more effective reduction of the inflammatory process when using the IL-1β receptor antagonist. The use of the IL-1β receptor antagonist (Anakinra) promotes a more effective restoration of impaired metabolic and antioxidant processes, which is confirmed by a significant decrease in MMP-2 concentration, iNOS expression, and nitrotyrosine levels, an increase in eNOS expression, and a decrease in lactate dehydrogenase activity, indicating a reduction in oxidative stress and ischemic disorders. The use of the IL-1β receptor antagonist provides a targeted normalization of the cytokine profile in the gingival crevicular fluid, which is manifested by a decrease in IL-1β and IL-6 levels and an increase in IL-10, is associated with improved clinical indicators, and has high prognostic significance (AUC = 0.91 for IL-1β), confirming the leading role of IL-1β-dependent mechanisms in the pathogenesis of periodontitis (387, 398).

All of this supports this possible promising strategy of drug blockade of IL-1β, which may offer new prospects for the treatment of periodontitis. We would also like to provide a balanced assessment of both the potential and limitations of IL-1 blockade therapy. **Potential for therapy**: high efficacy in inflammatory periodontal diseases; short latent period of therapeutic action; prevention of severe complications in chronic periodontitis, because IL-1 blockers effectively protect target organs from systemic inflammatory damage; safety for immunity, because IL-1 blockers do not cause generalized immunodeficiency and rarely lead to opportunistic infections. **Limitations**: Specificity of action does not always determine effectiveness if inflammation can be supported by other cytokines; long-term use may lead to masking of infections due to suppression of the production of endogenous pyrogens; with long-term use, there is some risk of bacterial invasions; with long-term use, the occurrence of metabolic and other adverse reactions is predicted. However, additional independent clinical studies are needed to confirm this. However, despite encouraging results from *in vitro* and *in vivo* experimental studies, clinical expectations regarding their efficacy are not always met, representing a major translational challenge. This discrepancy stems from several objective factors, including biological and species-specific differences, as well as the fact that experimental models frequently fail to account for comorbidities, environmental factors, dietary habits, occupational activities, and lifestyle factors (such as smoking). Furthermore, experimental models cannot replicate the full complexity of the molecular and biochemical mechanisms leading to periodontitis, nor can they capture the immense variability of the human periodontal microbiota (395). Improving translational continuity requires the implementation of multiple periodontitis models, the standardization of evidence-based assessment systems, a comprehensive multidisciplinary evaluation of outcomes, and the integration of novel information technologies (117, 119, 194, 207, 254). A limitation of the proposed model is that the clinical and preclinical validation of the suggested targeted IL-1β modulation strategy relies predominantly on the authors’ own research findings, owing to the limited number of independent studies on this topic. This underscores the need for further multicenter studies to confirm the reproducibility of the reported results.

## 8 Conclusions

To sum up, based on the analysis of the results of comprehensive molecular and biochemical studies on animal models and clinical laboratory studies, data on the subtle IL-1β-dependent links in the pathogenesis of chronic periodontitis have been expanded, and data on the reciprocity of IL-1β and endogenous cytoprotective factors (70 kDa heat shock proteins, the glutathione system, etc.) in the pathogenesis of this inflammatory disease of periodontitis have been revealed. Based on the analysis of open sources, it has been shown that the pharmacological blockade of IL-1β in periodontitis leads to the activation of endogenous cytoprotection, then to normalization of the NO system and the glutathione system, and finally to a decrease in oxidative stress, ischemia, and clinical symptoms of the disease. The prospects of a new strategy of drug blockade of IL-1β (inclusion in the complex drug therapy of IL-1β receptor blockers in different dosage forms and routes of administration) are observed, which may have new prospects in the treatment of periodontitis.

In our review for specialists and researchers of a broad medical and biological profile, we present a unified cohesive model of cytokine-dependent links in the pathogenesis of chronic periodontitis by covering various signaling pathways, relying on both primary and secondary sources. The review also theoretically substantiates promising target compounds for the drug treatment of chronic periodontitis, using the example of the use of Il-1B receptor antagonists. The proposed strategy of pharmacological targeted modulation has specificity as well as preclinical and clinical validation (although based on the example of our own research in the absence of other data). All this demonstrates a clear articulation of the novelty of the work and its difference from other similar reviews, because it does not repeat already well-established concepts but showcases innovative perspectives in the treatment of periodontitis. Thus, the results of experimental and clinical studies demonstrate a promising strategy for drug blockade of IL-1, which may open up new prospects for the treatment of periodontitis.
